# Engineering adaptive polysaccharide biomaterials: integrated biotechnology framework for regenerative medicine

**DOI:** 10.1186/s13036-026-00786-y

**Published:** 2026-10-23

**Authors:** Sameh S. Ali, Kashif Khan, Hend Elkahlawy Hassan, Asmaa Ali, Fatma Abd El Aziz, Michael Schagerl, Abd El-Raheem El-Shanshoury, Mahran Mohamed Abd El-Emam

**Affiliations:** 1 Biofuels Institute, School of The Environment and Safety Engineering, Jiangsu University, Zhenjiang, 212013 China; 2 Botany and Microbiology Department, Faculty of Science, Tanta University, Tanta, 31527 Egypt; 3 Department of Laboratory Medicine, School of Medicine, Jiangsu University, Zhenjiang, 212013 China; 4 Department of Functional and Evolutionary Ecology, University of Vienna, Djerassiplatz 1, Vienna, A-1030 Austria; 5 Department of Biochemistry and Molecular Biology, Faculty of Veterinary Medicine, Zagazig University, Zagazig, 44511 Egypt; 6 Department of Biochemistry, Faculty of Pharmacy, El Saleheya El Gadida University, El Saleheya El Gadida, 44813 Egypt

**Keywords:** Adaptive polysaccharide biomaterials, Biological engineering, Regenerative medicine, Biofabrication, Tissue engineering, Translational biomaterials

## Abstract

Natural polysaccharides are versatile building blocks for regenerative biomaterials because of their structural diversity, generally favorable biocompatibility and biodegradability, chemical tunability, and capacity for biologically relevant interactions. Advances in biotechnology and materials engineering have expanded their role from primarily structural matrices to multifunctional platforms that can influence cellular, immune, and biochemical processes during tissue repair. This review presents an integrated biological engineering framework linking polysaccharide source and molecular architecture with extraction and purification, chemical functionalization, crosslinking, advanced fabrication, biological recognition, and translational performance. Particular emphasis is placed on structure–function relationships, stereospecific glycan–receptor interactions, immune-instructive biomaterials, protein-mediated cell–material interactions, microbiome-responsive systems, and emerging artificial intelligence-assisted approaches for biomaterial design. The importance of material purity is also critically considered, particularly the potential for residual endotoxin contamination to confound interpretation of immunomodulatory responses and compromise reproducibility. Representative applications in skin, musculoskeletal, cardiovascular, neural, and other regenerative systems illustrate how material composition, scaffold architecture, biofunctionalization, and manufacturing strategy collectively influence biological performance. Finally, challenges associated with scalable manufacturing, batch-to-batch reproducibility, sterilization, storage stability, quality control, regulatory translation, and clinical implementation are examined. By integrating molecular design, biological mechanisms, fabrication, and translational requirements, this review provides engineering principles for the development of adaptive polysaccharide biomaterials and identifies priorities for advancing reproducible and clinically translatable regenerative technologies.

## Introduction

 Polysaccharides constitute a structurally diverse and biologically versatile class of natural polymers whose use has evolved from primarily structural materials to multifunctional components of regenerative scaffold systems [1, 2]. Derived from plant, animal, algal, and microbial sources, polysaccharides such as chitosan, alginate, hyaluronic acid, dextran, cellulose, chondroitin sulfate, and heparin have attracted sustained interest because of their generally favorable biocompatibility, biodegradability, structural diversity, and capacity for biologically relevant interactions [3–7]. Their molecular architectures contain reactive functional groups, including hydroxyl, carboxyl, sulfate, and amine moieties, that permit chemical modification and tunable control over physicochemical and biological properties [8]. These characteristics enable polysaccharides to serve not only as structural matrices but also as biointeractive materials capable of influencing cellular behavior, immune responses, and tissue remodeling.

Within regenerative medicine, polysaccharide-based scaffolds have gained increasing attention because they can reproduce selected physicochemical and biochemical features of the extracellular matrix (ECM) while providing tunable mechanical and biological functionality. Early applications relied largely on relatively simple hydrogels, membranes, and films, whereas contemporary systems increasingly incorporate multifunctional designs capable of supporting cell delivery, presenting therapeutic cues, and guiding tissue-specific regeneration [9, 10]. Advances in fabrication technologies, including electrospinning, three-dimensional (3D) printing, and micro- and nanofabrication, have further enabled control over scaffold architecture, porosity, spatial organization, and mechanical properties [11]. In parallel, injectable polysaccharide-based hydrogels are being investigated as minimally invasive platforms for localized delivery of cells, growth factors, drugs, and other bioactive agents, with the potential to provide sustained release and promote tissue integration [12, 13].

Beyond their structural roles, polysaccharide-based biomaterials can actively influence biological responses, particularly inflammatory and immune processes. Depending on their molecular structure, chemical modification, physical organization, and biological context, polysaccharides may interact directly or indirectly with cell-surface receptors and associated signaling pathways, including those involving cluster of differentiation 44 (CD44), Toll-like receptors (TLRs), and C-type lectin receptors [11, 14]. Such interactions can influence macrophage activation states, inflammatory signaling, angiogenesis, and tissue remodeling. Importantly, these responses should not be interpreted as fixed material properties because they are influenced by multiple interdependent parameters, including molecular weight, functional-group presentation, mechanics, degradation behavior, adsorbed proteins, and the local biological microenvironment. Emerging strategies further extend this concept toward microbiome-responsive biomaterials, in which microbial metabolism and metabolites such as short-chain fatty acids (SCFAs) can participate in host–material interactions and regenerative processes [15, 16].

In parallel, data-driven and artificial intelligence (AI)-assisted approaches are beginning to influence biomaterial design and optimization. Machine-learning methods can facilitate analysis of multidimensional relationships among composition, processing conditions, material properties, and biological responses, thereby supporting more efficient experimental design and candidate screening [17, 18]. These computational approaches complement emerging biofunctionalization strategies, including ligand engineering, spatially graded architectures, and incorporation of extracellular vesicles or other biologically active components, which can further enhance the instructive capacity of polysaccharide-based scaffolds [19].

Despite significant progress, existing reviews often address individual aspects of polysaccharide biomaterials separately. For example, Niziołek et al. [20] and Rumon et al. [21] primarily discuss hydrogel-based systems, whereas Matai et al. [22] emphasize fabrication and biofabrication technologies, and Salih et al. [23] focus on application-specific aspects. Although these studies provide valuable insights, relatively few reviews integrate molecular architecture, processing and purification, chemical modification, advanced fabrication, immune regulation, biological signaling, and translational considerations within a single engineering framework. Establishing relationships among these interconnected levels is important because decisions made during polysaccharide sourcing, processing, modification, and fabrication ultimately influence scaffold properties, host responses, and regenerative performance.

An additional consideration when interpreting the biological activity of natural polysaccharides is the potential contribution of process-derived impurities, particularly bacterial endotoxins. Natural and microbial polysaccharide preparations, as well as some commercially available materials, may contain residual lipopolysaccharide (LPS) or other microbial-associated contaminants when extraction and purification are insufficient. Because LPS is a potent activator of innate immune signaling, even relatively low contamination levels can alter macrophage and other immune-cell responses and thereby confound attribution of an observed inflammatory or immunomodulatory effect to the intrinsic chemistry of the polysaccharide. This issue is particularly important when apparently divergent pro-inflammatory and anti-inflammatory, or classically described M1- and M2-associated, responses are reported for materials from the same polysaccharide class. Lee et al. [24] further demonstrated that differences in chitosan physicochemical characteristics, including molecular weight and molecular-scale mechanical properties, can contribute to divergent immune responses, emphasizing the need to distinguish intrinsic structure-dependent activity from experimental confounders. Accordingly, studies evaluating polysaccharide-mediated immune responses should consider purification history, endotoxin burden, and appropriate controls and should, where relevant, employ validated endotoxin testing and adequately depyrogenated or endotoxin-controlled material preparations.

To address these interconnected challenges, this review presents an integrated biological engineering framework for the rational design and translation of adaptive polysaccharide biomaterials for regenerative medicine. Here, adaptive polysaccharide biomaterials refers to engineered polysaccharide-based systems whose physicochemical or biological functions can be tuned or can respond to relevant biochemical, cellular, mechanical, or microenvironmental cues during tissue repair. Rather than treating molecular design, processing, chemical modification, fabrication technologies, immune regulation, and regenerative applications as independent topics, the review examines how these processes collectively determine biomaterial performance and translational potential. Particular emphasis is placed on structure–function relationships, advanced biofabrication, immune-instructive and microbiome-responsive biomaterials, and emerging AI-assisted design strategies. The review further examines challenges associated with scalable biomanufacturing, reproducibility, quality control, regulatory translation, and clinical implementation. By integrating these dimensions within a unified framework, we aim to identify practical design principles and future opportunities for developing multifunctional and clinically translatable polysaccharide biomaterials.

## Structural and physicochemical foundations of polysaccharides

### Sources and classification of biomedical polysaccharides

**Fig. 1:**
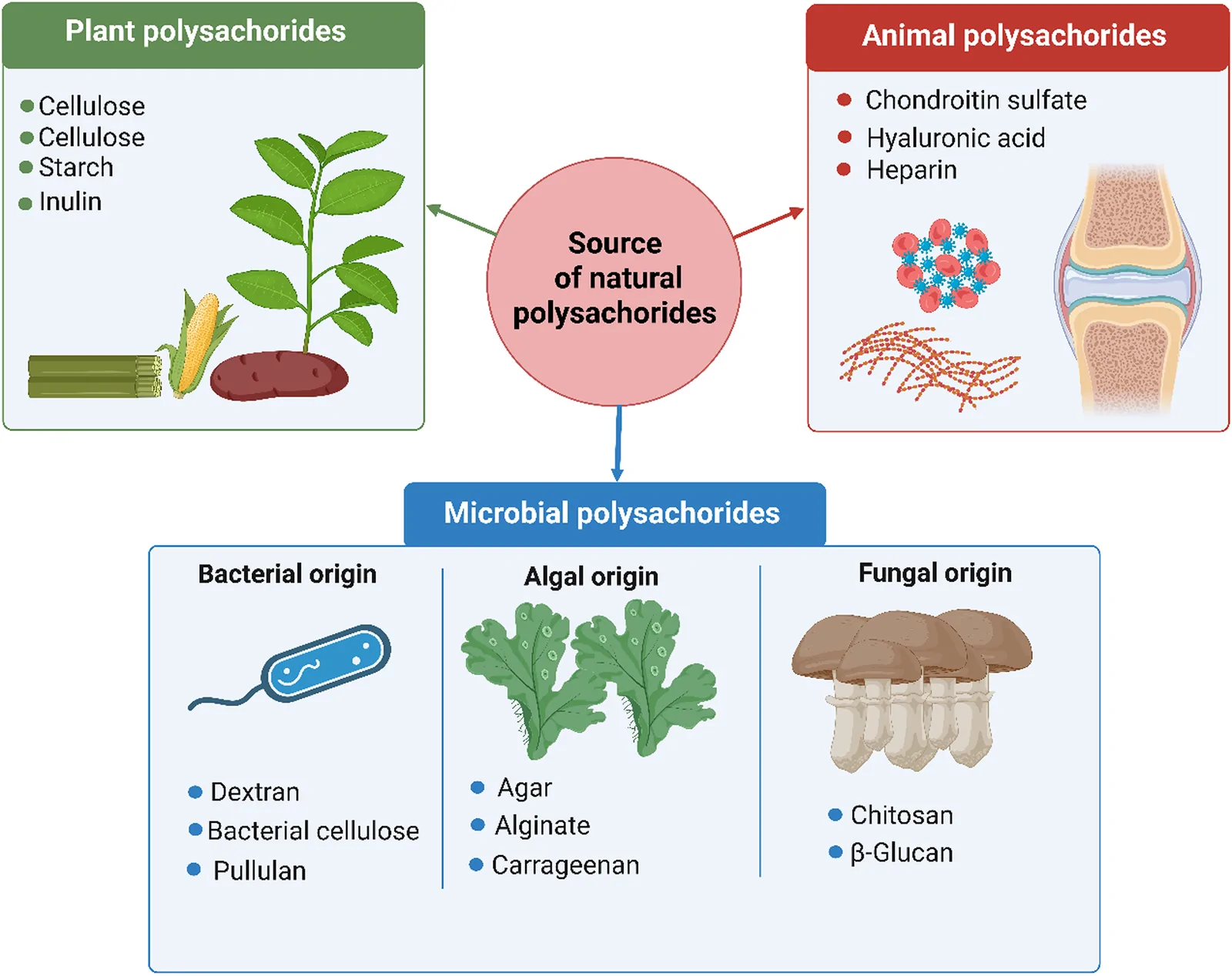
Biological sources and representative classes of polysaccharides used in regenerative biomaterials. Naturalpolysaccharides are grouped according to plant, animal, microbial, and marine/algal origin.Representative polymers are shown for each source category, together with their principal relevance tobiomedical scaffold design

Natural polysaccharides constitute a broad and heterogeneous class of biopolymers whose molecular and biological properties are strongly influenced by their biological origin. For biomedical applications, they can be broadly categorized as plant-derived, animal-derived, microbial-derived, and marine/algal-derived polysaccharides. Fungal polysaccharides may be considered within the microbial-derived group because of similarities in biosynthetic origin and fermentation-based production. This source-based classification is not merely taxonomic; biological origin influences monomer composition, glycosidic linkage patterns, branching, molecular weight, functional-group distribution, purity, degradation behavior, and biological activity, thereby affecting suitability for specific regenerative-medicine applications [25]. As summarized in Fig. 1, these source-dependent structural characteristics provide a useful basis for selecting polysaccharides according to the mechanical, chemical, and biological requirements of a particular biomedical application.

Plant-derived polysaccharides, including cellulose, hemicellulose, pectin, starch, and inulin, are among the most abundant and renewable biopolymers used in biomedical materials [26]. Their wide availability, generally favorable biocompatibility, renewability, and abundance of hydroxyl and carboxyl groups facilitate chemical modification and processing into scaffolds, hydrogels, films, fibers, and composite matrices. Depending on polymer structure and processing conditions, plant-derived polysaccharides can provide mechanical stability together with tunable solubility, degradation, and bioactivity. They are therefore frequently employed as structural matrices or reinforcing components in tissue-engineering constructs, particularly in applications in which mechanical integrity and sustainable material sourcing are important [27].

Animal-derived polysaccharides are represented predominantly by glycosaminoglycans, including hyaluronic acid, chondroitin sulfate, heparin, and keratan sulfate, which are integral components of native extracellular matrices [28, 29]. Chitin and its deacetylated derivative chitosan also constitute important animal-derived polysaccharides, although chitin and chitosan may additionally be obtained from fungal sources. Because many animal-derived polysaccharides structurally resemble ECM constituents, they can participate in hydration, cell–matrix interactions, growth-factor binding, cell migration, and tissue remodeling. These characteristics have supported their use in injectable hydrogels, cartilage-repair systems, wound-healing matrices, drug-delivery platforms, and immunomodulatory scaffolds [30].

Microbial-derived polysaccharides, including bacterial cellulose, dextran, gellan gum, xanthan gum, pullulan, and levan, constitute an important class of biomaterials that can be produced through controlled fermentation processes. Such production strategies can provide comparatively reproducible material characteristics and enable modulation of molecular properties through strain selection, culture conditions, and downstream processing. Among these polysaccharides, levan is a microbial exopolysaccharide composed predominantly of β-(2→6)-linked fructose residues, with structural branching and molecular characteristics that vary according to the producing microorganism and processing conditions. Its high-water solubility, hydration capacity, reported antioxidant activity, and cytocompatibility have supported its investigation in controlled-delivery systems, hydrogels, wound dressings, and other regenerative biomaterials [31–33]. Microbial polysaccharides more broadly have also been explored in wound dressings, injectable depots, bioinks, and 3D bioprinted scaffolds [34], while some microbial polysaccharides may participate in host–microbiome interactions relevant to emerging microbiome-responsive and immune-regulatory biomaterials [16, 35].

Chemical modification can further expand the biomedical utility of levan by addressing limitations of the native polymer, particularly its relatively low intrinsic viscosity and limited retention in wet environments. Methacrylate-functionalized levan derivatives have been photocrosslinked to form hydrogels with tunable mechanical properties, hydrolytic degradability, and cytocompatibility, demonstrating the potential of levan-based macromers as adaptable hydrogel platforms [36]. Taylan et al. [37] reported antioxidant and immunomodulatory activity for levan produced by *Leuconostoc mesenteroides* S81, further supporting its potential as a biologically active microbial polysaccharide [37]. Of particular relevance to regenerative medicine, Osman et al. [32] developed catechol-conjugated levan to improve adhesion under wet conditions. The resulting levan–catechol hydrogel exhibited strong adhesion to hydrated porcine skin, promoted rapid blood clotting, and accelerated healing of rat-skin incisions compared with untreated controls, supporting its potential for hemostatic and tissue-adhesive applications [32]. These studies illustrate how chemical functionalization can convert the favorable intrinsic characteristics of levan into application-specific biomaterial functions.

Marine- and algal-derived polysaccharides, obtained primarily from algae and seaweeds, include alginate, carrageenan, fucoidan, and agarose. These materials are distinguished by structural characteristics such as high anionic charge density, variable sulfation patterns, and ion-responsive gelation behavior [38–40]. Such properties enable high water retention, mild gelation conditions, and interactions with proteins and growth factors, making marine polysaccharides particularly attractive for hydrogel-based scaffolds, drug-delivery systems, wound-healing materials, bioinks, and immunomodulatory biomaterials. Their structural diversity also provides opportunities to tune mechanical performance, degradation, and biological signaling for tissue-specific regenerative applications.

### Molecular architecture and structure

The molecular architecture of polysaccharides governs their physicochemical behavior and underpins their performance as biomedical materials. Key structural parameters, including monosaccharide composition, glycosidic linkage type, chain conformation, molecular weight, branching density, and functional group distribution, collectively determine solubility, mechanical strength, degradation kinetics, and biological interactions. Understanding these structure–property relationships is essential for the rational design of polysaccharide-based scaffolds and provides a mechanistic basis for subsequent processing, crosslinking, and biofunctionalization strategies.

At the primary structural level, the identity and stereochemistry of constituent monosaccharides strongly influence polymer flexibility and hydration. Polysaccharides composed of neutral sugars, such as glucose-based cellulose and dextran, typically exhibit high hydrogen-bonding capacity and structural rigidity, whereas those containing uronic acids or amino sugars, such as alginate, hyaluronic acid, and chondroitin sulfate, possess increased chain flexibility and polyanionic character. The presence of charged groups enhances water uptake, osmotic swelling, and ion-mediated interactions, which are critical for hydrogel formation and viscoelastic behavior [28, 38].

Glycosidic linkage type and chain conformation further modulate mechanical and rheological properties. Linear β-(1→4)-linked polysaccharides, such as cellulose and chitin, form extended chain conformations stabilized by extensive intra- and intermolecular hydrogen bonding, resulting in high tensile strength and crystallinity. In contrast, α-linked or mixed-linkage polysaccharides, such as dextran and starch derivatives, exhibit greater flexibility and processability [41]. Branching architecture also plays a decisive role, as highly branched polysaccharides generally display increased solubility and chain mobility, whereas linear polymers favor ordered packing and enhanced mechanical robustness [42].

Molecular weight is another critical determinant of polysaccharide behavior in biomedical contexts. High-molecular-weight polysaccharides typically provide superior mechanical integrity and prolonged in vivo residence, whereas lower-molecular-weight variants exhibit enhanced diffusivity, faster degradation, and improved bioavailability [43]. For instance, variations in hyaluronic acid molecular weight are known to elicit distinct biological responses, influencing inflammation, angiogenesis, and cell migration [44, 45]. Controlled modulation of molecular weight through extraction, depolymerization, or biosynthesis therefore represents a powerful strategy for tuning scaffold performance.

Functional group chemistry is central to both intrinsic bioactivity and downstream material customization. Hydroxyl groups enable extensive hydrogen bonding and chemical derivatization, while carboxyl, sulfate, and amino groups impart charge-dependent interactions with ions, proteins, and cell receptors. Sulfated polysaccharides, such as heparin and fucoidan, exhibit strong affinity for growth factors and cytokines, enabling regulation of signaling pathways involved in angiogenesis, inflammation, and tissue remodeling [46, 47]. Amino-containing polysaccharides, notably chitosan, display pH-responsive behavior and intrinsic antimicrobial activity due to protonation of amine groups under physiological conditions [48].

These molecular features collectively influence degradation behavior, which is a critical parameter for regenerative applications. Polysaccharide degradation occurs via enzymatic or hydrolytic pathways and is governed by chain accessibility, crystallinity, functional group density, and crosslinking susceptibility. Highly ordered regions degrade more slowly, whereas amorphous or chemically modified domains are more susceptible to enzymatic cleavage [49]. Matching degradation kinetics with tissue healing rates is essential to ensure scaffold support during regeneration while minimizing chronic inflammation or foreign body responses. Importantly, molecular architecture not only dictates bulk material properties but also governs biological recognition at the cell–material interface. Polysaccharide conformation, charge distribution, and sulfation patterns influence protein adsorption, receptor binding, and downstream signaling pathways. Interactions with receptors such as CD44 and integrins are mediated by specific structural motifs rather than polymer identity alone, underscoring the need for architecture-informed material design [11, 14].

Collectively, these structure–property relationships establish a molecular framework that guides the rational selection and engineering of polysaccharides for biomedical use. By linking molecular architecture to mechanical, chemical, and biological behavior, this section provides a foundation for understanding how polysaccharides can be strategically tailored prior to processing and fabrication. While these structural characteristics are extensively discussed in previous literature, the present section emphasizes features that directly influence scaffold design, processing behavior, and biological performance.

### Intrinsic biocompatibility, biodegradability, and bioactivity

The widespread use of polysaccharides in biomedical applications is associated with their generally favorable biocompatibility, biodegradability, structural diversity, and capacity for biologically relevant interactions. Many natural polysaccharides share chemical or structural features with components of native extracellular matrices and glycosaminoglycan networks, supporting their use in regenerative biomaterials [50, 51]. However, biological compatibility should not be regarded as an inherent or universal property of all polysaccharides, because host responses are influenced by polymer source, molecular weight, chemical modification, degradation products, processing conditions, and material purity.

The biocompatibility of individual polysaccharides is strongly influenced by their molecular structure and physicochemical characteristics. Polysaccharides such as hyaluronic acid, chondroitin sulfate, heparin, and selected cellulose derivatives have been widely investigated because of their generally low cytotoxicity and their ability to support cellular and tissue interactions under appropriate formulation conditions [7, 10]. Their hydrophilic character promotes hydration of the material interface and can influence protein adsorption, cell adhesion, and matrix organization. Hyaluronic acid, for example, contributes to tissue hydration and interacts with cellular receptors involved in migration, inflammation, and matrix remodeling [52]. Chondroitin sulfate has likewise been associated with anti-inflammatory, antioxidant, antithrombotic, and immunomodulatory activities in different biological contexts [53–56]. Importantly, these biological responses depend on molecular characteristics and experimental context rather than representing fixed properties of the polysaccharide class.

Biodegradability represents another important advantage of many polysaccharide-based biomaterials. Degradation can occur through enzymatic, hydrolytic, or environmentally mediated processes, depending on the chemical structure of the polysaccharide and the biological environment [49]. Degradation kinetics are influenced by molecular weight, crystallinity, chain accessibility, functional-group density, crosslinking degree, and chemical modification. Highly ordered or crystalline structures, such as those found in cellulose and chitin, generally exhibit slower degradation than less ordered structures, whereas chemical modification and crosslinking can substantially alter degradation rates. Accordingly, degradation behavior should be tailored to the intended regenerative application so that material resorption is coordinated with tissue formation and remodeling. The biological compatibility of degradation products should also be evaluated for each formulation rather than assumed solely from the natural origin of the parent polymer [57].

Beyond structural compatibility and degradation, many polysaccharides can participate directly in biological signaling. Such bioactivity may arise through receptor-mediated recognition, regulation of ECM assembly, or interactions with growth factors, cytokines, and other signaling molecules. Hyaluronic acid, for example, interacts with receptors including CD44 and the receptor for hyaluronan-mediated motility (RHAMM), thereby influencing processes such as cell migration, proliferation, inflammatory signaling, and angiogenesis. Sulfated polysaccharides, including heparin and fucoidan, can bind and stabilize growth factors and other signaling proteins, thereby modifying their local availability and biological activity [46, 47]. These interactions enable appropriately designed polysaccharide matrices to function as biointeractive materials rather than merely passive carriers.

Importantly, polysaccharide bioactivity is not governed solely by nonspecific physicochemical characteristics such as charge, wettability, or hydrophilicity. Direct stereospecific recognition of defined carbohydrate structures by glycan-binding proteins represents a distinct mechanism through which polysaccharides can regulate biological responses. Monosaccharide composition, stereochemistry, glycosidic linkage, branching pattern, molecular weight, chain conformation, and spatial presentation of carbohydrate motifs can influence recognition by C-type lectin receptors, including Dectin-family receptors, as well as by galectins and Siglecs. Lee et al. [58] demonstrated that structurally distinct yeast-derived polysaccharides can elicit different immune responses through glycan-dependent recognition mechanisms, illustrating how differences in β-glucan and mannan-containing structures may result in divergent biological effects. Recognition of β-(1,3;1,6)-glucans by Dectin-1 is also influenced by polymer conformation and higher-order structural organization, emphasizing that receptor activation depends not simply on carbohydrate composition but also on molecular presentation and chain architecture. Choi et al. [59] further demonstrated the importance of specific carbohydrate motifs and stereochemical presentation in carbohydrate–protein recognition. Collectively, these findings indicate that precise glycan architecture is a fundamental determinant of receptor-mediated polysaccharide bioactivity [58, 59].

Glycan-mediated recognition may also contribute directly to regenerative processes. Galectin-dependent signaling, for example, can influence inflammatory regulation, stem-cell behavior, and tissue remodeling. Mukhtar et al. [60] reported that poly-(L-lactide-co-ε-caprolactone) scaffolds functionalized with modified rhamnogalacturonan-I potato pectin altered galectin-3-associated signaling and inflammatory gene expression in mesenchymal stem cells (MSCs), with corresponding effects on bone-regenerative responses. Such observations illustrate that the regenerative activity of polysaccharides may depend on specific sugar–lectin interactions rather than on generalized “natural polymer” effects alone. At the same time, direct glycan–receptor recognition should be distinguished from indirect interfacial mechanisms. Polysaccharide chemistry, charge, hydrophilicity, and topography can alter the adsorption, orientation, and conformation of proteins at the biomaterial surface, thereby creating an interfacial protein layer that subsequently regulates cell adhesion and immune-cell behavior. These protein-mediated effects can occur in parallel with direct receptor recognition but represent mechanistically distinct pathways. This distinction is particularly important when interpreting immunomodulatory responses.

Polysaccharide-mediated regulation of inflammatory responses can therefore arise through several interacting mechanisms. Certain chemically defined polysaccharides or derivatives have been reported to promote macrophage phenotypes associated with tissue repair and resolution of inflammation [11]. For example, Shen et al. [61] reported that sulfated chitosan promoted an M2-associated macrophage phenotype through the Interleukin-6/ Signal transducer and activator of transcription 6/Peroxisome proliferator-activated receptor gamma (IL-4/STAT6/PPARγ) pathway, increased chemokine ligand 22 (CCL22) production, and enhanced recruitment of regulatory T cells in a diabetic wound model. These findings illustrate the potential for polysaccharide chemistry to modulate endogenous immune pathways; however, such responses should be interpreted within the specific molecular, experimental, and disease context in which they were observed.

A critical consideration in interpreting these biological effects is the purity and manufacturing history of the polysaccharide preparation. Trace endotoxin contamination can activate TLR4-dependent inflammatory pathways and induce cytokine production independently of the intended polysaccharide–receptor interaction. This issue is particularly relevant to chitosan and microbial-derived polysaccharides, for which differences in extraction, purification, and endotoxin burden can contribute to apparently contradictory immune phenotypes. Lee et al. [62] highlighted the diverse and sometimes opposing immune responses reported for chitosan and demonstrated that physicochemical characteristics such as molecular weight and material properties can substantially influence interactions with immune cells. Lee et al. [24] further emphasized the dependence of nanochitin and nanochitosan immune activity on material characteristics, experimental conditions, and endotoxin status. Accordingly, claims that a polysaccharide is intrinsically pro-inflammatory, anti-inflammatory, or M2-promoting should be supported by adequate characterization of material purity, validated endotoxin testing, and, where appropriate, endotoxin-depleted or matched-control preparations.

Taken together, the biocompatibility, degradability, receptor-mediated bioactivity, and interfacial biological interactions of polysaccharides are determined by an interconnected set of molecular and processing parameters rather than by polymer identity alone. Molecular architecture, stereochemistry, molecular weight, chemical modification, degradation behavior, protein adsorption, receptor recognition, and material purity collectively influence biological performance. Understanding these relationships provides a mechanistic foundation for the rational chemical modification, crosslinking, fabrication, and biofunctionalization strategies discussed in subsequent sections.

## Processing, modification, and crosslinking strategies

This section provides a comprehensive overview of key methods, including extraction and purification, chemical modification, gelation, and crosslinking, highlighting how these processes influence polysaccharide bioactivity, mechanical properties, and suitability for tissue engineering, drug delivery, and regenerative medicine.

### Extraction, purification, and molecular weight control

The extraction and purification of polysaccharides are foundational processing steps that determine material purity, molecular integrity, structural characteristics, bioactivity, and suitability for biomedical applications. Variations introduced during these stages can influence subsequent chemical modification, gelation behavior, mechanical performance, degradation, and biological responses. Accordingly, extraction and purification strategies should be selected not only to maximize yield but also to preserve relevant structural features, minimize process-derived contaminants, and support reproducible production of biomedical-grade polysaccharides. Extraction methods are largely determined by the biological source of the polysaccharide and the required balance among extraction efficiency, structural preservation, processing time, and scalability. Hot-water extraction (HWE) remains widely used because of its operational simplicity and compatibility with diverse biological sources [63], although prolonged exposure to elevated temperatures may promote partial depolymerization or loss of thermolabile structural features. Ultrasound-assisted extraction (UAE) enhances mass transfer and solvent penetration through cavitation, potentially improving extraction efficiency and reducing processing time [64]. However, excessive ultrasound intensity may also induce polymer-chain disruption. Microwave-assisted extraction (MAE) similarly accelerates mass transfer through rapid volumetric heating but requires optimization of temperature, exposure time, and microwave power to minimize polysaccharide degradation [65]. Enzyme-assisted extraction (EAE) provides a comparatively mild and selective approach by degrading surrounding cellular or matrix components while preserving target polysaccharide structures [66]. Chemical extraction using acidic or alkaline conditions remains useful for structurally resistant biomass, although extensive neutralization and downstream purification may be required to remove residual chemicals and limit structural alteration [64].

Following extraction, purification is required to remove proteins, pigments, salts, nucleic acids, residual processing reagents, low-molecular-weight compounds, and other impurities that may alter material performance or biological responses. Alcohol precipitation and salting-out methods are commonly used for initial concentration and fractionation because of their relative simplicity and scalability [66]. Membrane-based methods, particularly ultrafiltration and diafiltration, can provide molecular-weight-based fractionation while simultaneously reducing soluble contaminants. For applications requiring higher purity or narrower molecular-weight distributions, chromatographic techniques, including ion-exchange and size-exclusion chromatography, provide greater selectivity according to molecular charge or size [67], although their cost, processing time, and scale-up requirements may limit their use in bulk production.

For regenerative medicine, injectable systems, and implantable biomaterials, purification must additionally address pyrogenic and endotoxin contamination. Endotoxins, particularly LPS originating from Gram-negative bacteria, can activate innate immune pathways and thereby confound interpretation of the intrinsic biological activity of polysaccharides. Consequently, biomedical purification workflows should incorporate validated endotoxin-reduction strategies rather than relying solely on conventional protein, salt, or pigment removal [68]. Depending on the physicochemical characteristics of the target polysaccharide, suitable approaches may include repeated ultrafiltration/diafiltration, selective surfactant-phase extraction, adsorption-based procedures, and appropriately designed chromatographic purification. Importantly, the selected procedure should be validated both for endotoxin reduction and for retention of the molecular structure and biological functionality of the target polysaccharide [69].

Endotoxin control should be accompanied by validated bacterial endotoxin testing, such as the Limulus amebocyte lysate (LAL) assay or an appropriate recombinant reagent-based assay [70]. Endotoxin acceptance limits should not be interpreted as a single universal mass-based threshold applicable to all polysaccharide biomaterials; rather, the acceptable endotoxin burden depends on the intended route of administration, product type, dose, and applicable regulatory specification. Therefore, injectable, implantable, and other clinically intended polysaccharide materials should be evaluated against endotoxin acceptance criteria appropriate to their intended use and regulatory classification. Reporting the analytical method, sample preparation, detection limit, and measured endotoxin burden is also important for comparing biological studies and for distinguishing intrinsic polysaccharide-mediated responses from contaminant-induced immune activation [70, 71].

The importance of balancing endotoxin removal with preservation of polysaccharide structure and function has been investigated. Adam et al. [72] compared several approaches for removing residual LPS from microbial exopolysaccharides and found that some conventional purification procedures provided limited endotoxin removal or impaired polysaccharide bioactivity. In contrast, low-temperature phase partitioning using Triton X-114 produced substantial reduction in LAL activity while better preserving biological activity, illustrating the potential value of selective surfactant-phase extraction. Ultrafiltration has also been successfully incorporated into polysaccharide recovery and downstream-processing workflows, while de Vries et al. [73] demonstrated that integrated membrane-based downstream processing could produce polysaccharide preparations with low residual protein, deoxyribonucleic acid (DNA), and endotoxin levels. For hyaluronic-acid-based biomedical products, Baeva et al. [74] further demonstrated the applicability of kinetic chromogenic LAL testing for evaluating residual bacterial endotoxin. Together, these studies emphasize that extraction yield alone is insufficient as a measure of biomedical process quality; purification performance should also be assessed in terms of chemical purity, endotoxin burden, molecular integrity, and retention of biological function. Your present manuscript contains these individual examples but currently presents them as a long sequence of study descriptions.

Control over molecular weight is another important outcome of extraction and purification because molecular-weight distribution influences solubility, viscosity, diffusion, degradation kinetics, mechanical behavior, and biological interactions. Higher-molecular-weight polysaccharides can provide increased viscosity, mechanical stability, and prolonged residence under appropriate conditions, whereas lower-molecular-weight fractions generally exhibit greater diffusivity and faster degradation [43]. Molecular weight can be tailored using controlled hydrolysis, enzymatic depolymerization, membrane fractionation, and related processing strategies. However, the desired molecular-weight range should be selected according to the intended biological function rather than assuming that either high- or low-molecular-weight material is universally preferable.

Overall, selection of extraction and purification methods should consider polysaccharide source, required structural integrity, intended biological function, purity requirements, scalability, and regulatory expectations. Mild extraction procedures may better preserve sensitive structural motifs, whereas more intensive chemical or thermal methods can improve recovery from resistant biomass at the expense of greater downstream purification requirements. Similarly, preliminary concentration methods such as alcohol precipitation may be appropriate during bulk processing, whereas membrane separation, chromatography, and validated endotoxin-control procedures become increasingly important for biomedical-grade materials. Table 1 summarizes representative extraction and purification methods for biomedical polysaccharides.

**Table 1:** Extraction and purification methods for biomedical polysaccharides

Method	Principle	Advantages	Limitations	Biomedical purity/depyrogenation considerations	Polysaccharide(s)	Biological source	References
HWE	Solubilization and extraction using heated water	Simple; widely applicable; limited requirement for specialized reagents	Relatively low selectivity; prolonged heating may cause depolymerization or loss of heat-sensitive structures	Requires subsequent removal of proteins, pigments, salts, microbial contaminants, and potential endotoxins before biomedical use	Chitin; fucoidan	Cephalothorax/crustacean material; *Saccharina japonica*	[64, 75, 76]
UAE	Acoustic cavitation enhances solvent penetration and mass transfer	Reduced extraction time; can improve extraction efficiency	Excessive ultrasound intensity may disrupt polymer chains or alter molecular weight	Should be followed by appropriate purification; endotoxin burden should be evaluated for biomedical applications	Alginate	*Sargassum binderi*	[77]
MAE	Microwave-induced volumetric heating accelerates extraction and mass transfer	Rapid processing; improved extraction efficiency under optimized conditions	Excessive temperature or exposure may promote polymer degradation	Rapid extraction does not remove pyrogenic contaminants; downstream purification and endotoxin testing remain necessary	Chitosan; ulvan; fucoidan	*Metapenaeus monodon*, *Penaeus indicus*; *Ulva ohnoi*, *Ulva meridionalis*; *Fucus vesiculosus*, *Undaria pinnatifida*	[65, 78–81]
EAE	Enzymatic degradation of surrounding cellular or matrix components facilitates polysaccharide release	Mild conditions; improved selectivity; may preserve sensitive structural motifs	Enzyme cost; additional removal of residual enzymes/proteins may be required	Residual enzymes, proteins, microbial components, and endotoxins should be removed and assessed using validated quality-control procedures	Chitin; hyaluronic acid	Litopenaeus vannamei; Aetobatus narinari	[82–84]
Chemical extraction	Acidic or alkaline treatment disrupts biological matrices and releases polysaccharides	Effective for resistant biomass; adaptable to diverse raw materials	Possible depolymerization or chemical modification; requires neutralization and extensive washing	Residual acid/alkali, salts, and endotoxins should be controlled before biomedical application	Chitin	Squid pens and crustacean-derived biomass	[85, 86]
Alcohol precipitation	Reduction of polysaccharide solubility by addition of alcohol	Simple; inexpensive; scalable; useful for concentration and preliminary fractionation	Limited selectivity; possible co-precipitation of impurities and product loss	Generally insufficient as a stand-alone biomedical purification step; further purification and endotoxin control may be necessary	Dextran; pullulan	Plant/fungal-derived preparations, including Angelica sinensis, Codonopsis pilosula, Dendrobium officinale, Ligusticum wallichii, Panax notoginseng, and Ganoderma sinensis	[87]
Ultrafiltration/diafiltration	Membrane separation based primarily on molecular size	Scalable; useful for concentration, fractionation, buffer exchange, and removal of soluble impurities	Membrane fouling; incomplete removal of contaminants of similar size	Useful component of endotoxin-reduction workflows but should not automatically be considered a stand-alone depyrogenation procedure; performance requires validation	Hyaluronic acid; microbial EPS	Streptococcus zooepidemicus; microbial fermentation preparations	[88, 89]
Ion-exchange/size-exclusion chromatography	Separation according to charge and/or molecular size	High selectivity and purity; enables separation of polysaccharide fractions	Costly; labor- and time-intensive; scale-up may be challenging	Can remove low-molecular-weight and charged contaminants and contribute to endotoxin reduction; recovery and endotoxin clearance should be experimentally validated	OAP-1A and other fractionated polysaccharides	Ocimum albumseed	[90, 91]
Surfactant-phase extraction	Partitioning of LPS into a detergent-rich phase	Can substantially reduce endotoxin burden under relatively mild conditions	Residual surfactant must be removed; possible product loss or altered activity; process requires validation	Particularly relevant for endotoxin reduction from microbial polysaccharide preparations; both residual detergent and endotoxin should be quantified	Microbial EPS	Klebsiellasp. EPS	[92]
Salting out	Reduced polysaccharide solubility through increased ionic strength	Simple; inexpensive; useful for concentration	Limited selectivity; possible product loss and residual salts	Residual salts/ions should be removed and verified before biomedical use; additional purification is generally required	β-Glucan	Oat ( Avena sativa)	[93]
Metal-ion precipitation/coordination	Selective complexation or precipitation using metal ions	Can provide selective polysaccharide recovery	Possible metal-ion contamination; requires additional purification	Residual metal ions should be quantified and removed to acceptable levels; method is generally insufficient alone for biomedical-grade purification	Galactoglucomannan	Kiwifruit ( Actinidia deliciosa)	[94, 95]

EAE, enzyme-assisted extraction; EPS, exopolysaccharide(s); HWE, hot-water extraction; LAL, Limulus amebocyte lysate; LPS, lipopolysaccharide; MAE, microwave-assisted extraction; OAP-1A, polysaccharide fraction isolated from Ocimum album seed; UAE, ultrasound-assisted extraction

### Chemical functionalization strategies

Native polysaccharides often exhibit limited mechanical strength, processability, and functional specificity in their unmodified form. Chemical functionalization therefore represents a central strategy for expanding their applicability in biomedical engineering by enabling precise control over solubility, charge density, crosslinking behavior, degradation kinetics, and biological interactions. Through selective modification of hydroxyl, carboxyl, amino, and sulfate groups, polysaccharides can be transformed into adaptable platforms for hydrogel formation, scaffold fabrication, and bioactive delivery systems [91]. Importantly, these modifications are typically performed under mild conditions to preserve backbone integrity and intrinsic biocompatibility. Chemical functionalization strategies can be broadly categorized into classical derivatization approaches, which primarily modulate charge and reactivity, and modern chemoselective approaches, which introduce polymerizable or orthogonal functional groups [96]. The selection of modification strategy depends on the intended application, required mechanical properties, degradation profile, and compatibility with subsequent crosslinking and fabrication processes.

#### Oxidation, sulfation, and carboxylation

Classical derivatization strategies such as oxidation, sulfation, and carboxylation remain widely used due to their simplicity, scalability, and effectiveness in modulating polysaccharide properties. Oxidation introduces reactive aldehyde or carboxyl groups while preserving the overall polymer backbone. Periodate oxidation selectively cleaves vicinal diols to generate dialdehyde polysaccharides capable of forming covalent bonds through Schiff-base reactions [97]. This modification enhances hydrophilicity and degradation susceptibility, while also enabling crosslinking without the need for toxic reagents. In addition, oxidation can disrupt crystalline regions, increasing chain flexibility and enzyme accessibility, thereby accelerating biodegradation [98]. Sulfation introduces negatively charged sulfate groups that significantly alter electrostatic interactions and biological functionality. Sulfated polysaccharides exhibit strong affinity for growth factors, cytokines, and ECM proteins, enabling stabilization and localized presentation of signaling molecules [47]. This modification is widely used to mimic glycosaminoglycan-like behavior; however, the degree and pattern of sulfation must be carefully controlled, as they directly influence anticoagulant activity and immune responses [46]. Carboxylation, including carboxymethylation, increases hydrophilicity, solubility, and ion-binding capacity. The introduction of carboxyl groups enhances interaction with multivalent ions, facilitating ionic crosslinking and hydrogel formation [88]. It also enables pH-responsive behavior and provides reactive sites for further functionalization. Collectively, these classical strategies offer versatile and scalable routes for tuning charge density, reactivity, and degradation behavior prior to scaffold fabrication.

#### Methacrylation and click-chemistry approaches

Modern chemoselective approaches, including methacrylation and click chemistry, enable precise and controllable functionalization of polysaccharides with high spatial and chemical specificity. Methacrylation introduces polymerizable vinyl groups onto polysaccharide backbones, rendering them photo-crosslinkable under ultraviolet (UV) or visible light in the presence of suitable photoinitiators [99]. Methacrylated derivatives of polysaccharides such as hyaluronic acid, dextran, and chitosan allow rapid, spatially controlled network formation with tunable mechanical properties and degradation profiles [100, 101]. These materials are particularly advantageous for injectable systems, in situ gelation, and 3D bioprinting, where crosslinking must occur under mild, cell-compatible conditions. Click-chemistry approaches provide highly efficient, chemoselective reactions with minimal byproducts and high reaction fidelity. Common reactions include azide–alkyne cycloaddition, thiol–ene coupling, and Diels–Alder cycloadditions [102]. These strategies enable modular assembly of polysaccharide networks and facilitate orthogonal functionalization with peptides, growth factors, or nanoparticles, allowing precise control over functional group density and material composition. An important advantage of methacrylation and click-based strategies is their compatibility with advanced fabrication technologies. Functionalized polysaccharides can be integrated into photopatterning, microfabrication, and biofabrication workflows, enabling spatial control over mechanical properties, porosity, and bioactivity. Furthermore, these approaches support the development of dynamic and stimuli-responsive systems through incorporation of cleavable or reversible linkages [103]. While this section outlines chemical functionalization strategies, their direct implications for mechanical properties, degradation behavior, and biological performance are discussed in the following section.

### Impact of chemical modification on biological and mechanical performance

Chemical modification plays a central role in transforming naturally derived polysaccharides into tunable systems suitable for biomedical applications. Although native polysaccharides are inherently biocompatible and biodegradable, their unmodified forms often lack the mechanical stability, degradation control, and functional specificity required for advanced tissue engineering [104]. Functionalization enables precise regulation of these properties by modulating intermolecular interactions, crosslinking density, and cell–material interfaces. From a mechanical perspective, chemical modification governs network formation, stiffness, elasticity, and structural integrity. Adjusting functional group density and degree of substitution allows polysaccharide systems to span a wide mechanical range, from soft viscoelastic matrices suitable for neural or dermal tissues to more robust constructs capable of supporting load-bearing environments [105]. These mechanical properties arise from chemistry-driven control of crosslink density and molecular mobility, providing a foundation for tailoring scaffold performance prior to fabrication.

Chemical modification also regulates degradation behavior, a critical parameter in regenerative applications. Increased crosslinking density generally slows enzymatic and hydrolytic degradation, whereas the introduction of hydrophilic or labile groups enhances water uptake and enzyme accessibility [106]. This tunability allows scaffold degradation rates to be matched with tissue regeneration processes, ensuring temporary mechanical support while facilitating gradual matrix replacement.

Beyond mechanical effects, chemical modification substantially alters biological interactions at the cell–material interface. Surface chemistry and functional group composition influence protein adsorption, ligand presentation, and receptor engagement, thereby regulating cell adhesion, proliferation, migration, and differentiation. Sulfated polysaccharides, for example, exhibit enhanced affinity for growth factors and cytokines, enabling their sequestration and localized presentation within the matrix microenvironment. For example, heparan-sulfate chains borne by the cell surface proteoglycans are required to mediate signals of heparin-binding growth factors such as fibroblast growth factors (FGFs), heparin-binding-epidermal growth factor (HB-EGF), or vascular endothelial growth factor (VEGF) [107]. Other functional groups provide reactive sites for the conjugation of peptides, ECM motifs, or bioactive molecules, allowing biochemical cues to be incorporated without compromising the intrinsic biocompatibility of the polysaccharide backbone.

Chemical modification also impacts immune responses, even when immunomodulation is not explicitly targeted. Variations in charge density, hydrophilicity, and surface chemistry influence protein corona formation, macrophage behavior, and cytokine signaling [108]. While controlled modification can improve biointegration, excessive or poorly regulated functionalization may introduce cytotoxicity or unintended immune activation. Therefore, careful optimization of modification conditions is essential to balance functionality with biological safety. Importantly, the effects of chemical modification are highly interconnected. Mechanical stiffness can influence stem cell fate, degradation kinetics affect immune cell recruitment and matrix remodeling, and functional group density simultaneously governs crosslinking behavior and receptor-mediated signaling [109]. These coupled relationships highlight the need for integrated design strategies that consider mechanical, chemical, and biological properties simultaneously rather than independently.

Overall, chemical functionalization defines the property space within which polysaccharide biomaterials operate. By establishing mechanical strength, degradation profiles, and biological interactions at the molecular level, modification strategies provide the foundation for subsequent crosslinking, fabrication, and biofunctional design. In this context, chemistry serves as a key determinant of scaffold performance and guides the development of application-specific biomaterials.

### Gelation and crosslinking mechanisms

Gelation and crosslinking are central processes that convert soluble polysaccharide chains into 3D networks capable of retaining large amounts of water while maintaining mechanical integrity. These networks constitute the structural basis of polysaccharide-based hydrogels and scaffolds, providing a hydrated microenvironment that closely resembles native extracellular matrices. The nature of the crosslinking mechanism directly influences network architecture, mechanical strength, viscoelastic behavior, degradation kinetics, and biological compatibility. Accordingly, understanding gelation mechanisms is essential for rational scaffold design and for selecting appropriate processing and downstream fabrication routes in regenerative medicine. Polysaccharide gelation can be broadly classified into physical, chemical, and enzymatic or dynamic mechanisms [110, 111]. Physical gelation relies on non-covalent interactions such as ionic interactions, hydrogen bonding, and supramolecular assembly, enabling reversible network formation under mild conditions. Chemical crosslinking introduces covalent bonds that enhance mechanical stability and long-term durability, while enzymatic and dynamic systems provide bioinspired alternatives characterized by high specificity, cytocompatibility, and the ability to support in situ gelation and adaptive remodeling. In practice, these mechanisms are frequently combined in hybrid systems to balance competing requirements, such as injectability and self-healing behavior versus sustained mechanical support. For example, chitosan–gellan gum hydrogel scaffolds formed through polyelectrolyte complexation demonstrate how blending oppositely charged polysaccharides can modulate scaffold structure and mechanical performance for tissue engineering applications [112].Similarly, composite hydrogels based on gellan gum and carboxymethyl chitosan that incorporate both ionic (physical) and Schiff-base (chemical) crosslinking have been reported to exhibit enhanced mechanical integrity together with sustained drug release capabilities for wound healing applications [113]. These examples highlight how hybrid crosslinking strategies can integrate the advantages of multiple gelation mechanisms to achieve tunable structural and functional properties in polysaccharide-based biomaterials.

#### Physical crosslinking and supramolecular assembly

Physical crosslinking refers to the formation of polysaccharide networks through reversible, non-covalent interactions, including hydrogen bonding, ionic interactions, hydrophobic associations, and van der Waals forces. These interactions enable spontaneous or stimulus-responsive gelation under mild conditions, making physically crosslinked hydrogels particularly attractive for cell encapsulation, injectable systems, and in situ forming scaffolds [114]. Because no covalent bonds are formed, physically crosslinked networks often exhibit dynamic behavior, self-healing capacity, and responsiveness to environmental cues such as pH, temperature, or ionic strength.

Hydrogen bonding is one of the most common driving forces for physical gelation in polysaccharides rich in hydroxyl groups, such as cellulose, pectin, carrageenan, and dextran. Interchain hydrogen bonding promotes chain association and network formation, resulting in soft hydrogels with moderate mechanical strength. For example, Amidated pectin contains a high level of amide groups that promote stronger hydrogen bonding, leading to quicker gel formation and improved gel stability [115]. Although individual hydrogen bonds are weak, their collective contribution can stabilize supramolecular assemblies and support reversible gelation. These systems are particularly suitable for applications requiring dynamic remodeling or rapid dissolution under physiological conditions [116].

Ionic crosslinking represents a more robust form of physical gelation and is widely employed for anionic polysaccharides containing carboxyl or sulfate groups. Alginate is the most well-known example, forming hydrogels through coordination of divalent cations such as calcium or magnesium with guluronic acid residues, a process often described by the “egg-box” model. Ionically crosslinked alginate hydrogels form rapidly under mild conditions, preserve cell viability, and allow tunable mechanical properties through control of ion concentration and exposure time [117]. Similar ionic interactions have been exploited in pectin, carrageenan, and gellan gum systems [118], where multivalent ions induce network formation and enhance gel stiffness.

Polyelectrolyte complexation is another important physical crosslinking mechanism, occurring through electrostatic interactions between oppositely charged polysaccharides. For example, complexes formed between cationic chitosan and anionic polysaccharides such as alginate, gellan gum, or hyaluronic acid yield hydrogels with enhanced mechanical stability and tunable porosity [119, 120]. These complexes can be formed without chemical crosslinkers, reducing cytotoxicity risks while allowing control over network properties by adjusting polymer ratios, pH, or ionic strength. Polyelectrolyte complexation also enables the incorporation of bioactive molecules through electrostatic binding.

Hydrophobic interactions and chain entanglement further contribute to supramolecular assembly in certain polysaccharide systems, particularly those that have been partially modified or blended with amphiphilic components. Temperature-responsive polysaccharides and derivatives can undergo sol–gel transitions driven by changes in hydrophobic association, enabling injectable formulations that gel upon exposure to physiological conditions [121]. Physical entrapment of cells, proteins, or drugs within these networks can be achieved during gel formation, preserving biological activity due to the absence of harsh chemical reagents.

Despite their advantages, physically crosslinked hydrogels generally exhibit lower mechanical strength and long-term stability compared to covalently crosslinked systems. Network dissociation under dilution or ionic exchange can lead to premature degradation or loss of structural integrity [122]. Consequently, physical crosslinking is often combined with chemical or enzymatic strategies to form hybrid networks that retain dynamic behavior while achieving sufficient mechanical robustness. The design considerations governing such hybrid systems are discussed in later sections.

#### Chemical crosslinking strategies

Chemical crosslinking involves the formation of covalent bonds between polysaccharide chains, resulting in permanent 3D networks with enhanced mechanical strength, structural stability, and resistance to dilution or ionic exchange. Compared to physically crosslinked systems, chemically crosslinked hydrogels generally exhibit higher stiffness, longer degradation times, and improved durability under physiological conditions. These features make chemical crosslinking particularly suitable for applications requiring sustained mechanical support, prolonged implantation, or controlled degradation profiles [123]. Covalent network formation is typically achieved through reactions between functional groups naturally present on polysaccharide backbones such as hydroxyl, carboxyl, and amino groups, or those introduced via chemical modification. Common chemical crosslinking approaches include aldehyde-mediated reactions, carbodiimide coupling, genipin crosslinking, and photoinitiated polymerization. Each method offers distinct advantages and limitations with respect to reaction efficiency, cytocompatibility, and control over network architecture.

Aldehyde-based crosslinking relies on reactions between aldehyde groups and primary amines to form Schiff-base linkages. This strategy is often enabled through prior oxidation of polysaccharides, such as periodate oxidation, to generate dialdehyde functionalities. Aldehyde-mediated crosslinking proceeds under mild aqueous conditions and allows injectable or in situ gel formation [124]. However, the stability of Schiff-base bonds and the potential cytotoxicity associated with residual aldehyde groups require careful control of reaction conditions and degree of oxidation. Carbodiimide chemistry, most commonly employing 1-ethyl-3-(3-dimethylaminopropyl) carbodiimide (EDC) and N-hydroxysuccinimide (NHS) systems, facilitates the formation of amide bonds between carboxyl and amino groups without introducing permanent crosslinkers into the final material [125]. This zero-length crosslinking strategy has been widely applied to polysaccharides such as hyaluronic acid, chitosan, and alginate to enhance mechanical integrity while maintaining biocompatibility. Although carbodiimide-mediated crosslinking provides stable covalent networks, it typically requires post-reaction purification to remove residual reagents and is less suited for in situ gelation.

Genipin, a naturally derived crosslinker extracted from Gardenia jasminoides, has emerged as a biocompatible alternative to conventional chemical crosslinkers such as glutaraldehyde. Genipin reacts primarily with amino groups, forming stable crosslinks while exhibiting significantly lower cytotoxicity [126]. Genipin-crosslinked polysaccharide networks display improved elasticity, reduced swelling, and enhanced resistance to enzymatic degradation, making them attractive for soft tissue engineering applications. Nevertheless, slower reaction kinetics and characteristic blue coloration may limit its use in certain contexts. Photochemical crosslinking represents a powerful chemical strategy that enables spatial and temporal control over network formation. In this approach, polysaccharides functionalized with photo-reactive groups such as methacrylates or acrylates are crosslinked upon exposure to UV or visible light in the presence of photoinitiators. Photopolymerization allows rapid gelation, precise patterning, and compatibility with advanced fabrication techniques such as photolithography and bioprinting [127]. Mechanical properties can be finely tuned by adjusting light intensity, exposure time, and degree of functionalization. However, potential phototoxicity and limited light penetration in thick constructs necessitate careful optimization.

Chemical crosslinking strategies provide robust and customizable networks but must be carefully balanced against cytocompatibility and processing constraints. Excessive crosslinking density may restrict cell infiltration, impair nutrient diffusion, or delay degradation, whereas insufficient crosslinking compromises mechanical stability. As a result, chemically crosslinked systems are often combined with physical or enzymatic mechanisms to form hybrid networks that integrate strength with dynamic biological behavior. Such integrative approaches are increasingly adopted to meet the complex demands of regenerative scaffolds.

#### Enzymatic and dynamic crosslinking systems

Enzymatic and dynamic crosslinking systems offer biologically inspired alternatives to conventional chemical crosslinking by enabling highly specific, cytocompatible, and often reversible network formation under physiological conditions. These strategies are particularly advantageous for applications involving cell encapsulation, injectable hydrogels, and in situ gelation, where harsh reaction conditions or toxic reagents must be avoided. By leveraging enzyme-catalyzed reactions or reversible covalent and supramolecular bonds, polysaccharide-based networks can achieve dynamic remodeling behavior that more closely mimics native ECM environments [128].

Enzymatic crosslinking relies on naturally occurring enzymes to catalyze bond formation between functional groups on polysaccharide chains or between polysaccharides and complementary polymers. Horseradish peroxidase (HRP) is among the most widely used enzymes for hydrogel formation, catalyzing the oxidative coupling of phenol- or tyramine-modified polysaccharides in the presence of low concentrations of hydrogen peroxide [129]. HRP-mediated crosslinking proceeds rapidly under mild aqueous conditions and enables precise temporal control over gelation kinetics by adjusting enzyme or peroxide concentration. This approach has been extensively applied to polysaccharides such as hyaluronic acid, dextran, and alginate derivatives, yielding hydrogels with tunable stiffness and excellent cytocompatibility.

Transglutaminase-mediated crosslinking represents another enzymatic strategy, catalyzing covalent bond formation between glutamine and lysine residues. For instance, transglutaminase-mediated crosslinking of polysaccharide-rich composite networks has been shown to enhance scaffold elasticity while supporting cell adhesion and proliferation [130]. Enzymatic specificity minimizes off-target reactions and allows gelation to occur selectively in complex biological environments, making these systems attractive for injectable and minimally invasive therapies. Beyond enzymatic approaches, dynamic crosslinking systems based on reversible covalent bonds have gained increasing attention for their ability to impart self-healing, stress relaxation, and adaptive mechanical behavior. Schiff-base reactions between aldehyde and amine groups, although sometimes categorized under chemical crosslinking, are often considered dynamic systems due to their reversible nature under physiological conditions. These imine bonds allow hydrogels to undergo network rearrangement, enabling injectability and self-repair while maintaining structural cohesion [131]. Similarly, boronate ester formation between diols and boronic acids provides pH-responsive and reversible crosslinking, offering opportunities for stimuli-responsive polysaccharide hydrogels [132].

Host-guest supramolecular interactions also contribute to dynamic network formation when polysaccharides are functionalized with complementary binding motifs. Cyclodextrin-based inclusion complexes, for example, have been widely used to form reversible crosslinks via non-covalent host–guest interactions, yielding hydrogels with shear-thinning and self-healing properties [133]. These systems enable repeated network disruption and reformation without loss of integrity, closely resembling the dynamic behavior of natural extracellular matrices. The primary advantage of enzymatic and dynamic crosslinking systems lies in their ability to balance mechanical stability with biological adaptability. Dynamic bond exchange allows networks to dissipate stress, accommodate cell migration, and respond to biochemical cues, while enzymatic specificity ensures minimal cytotoxicity and high reaction efficiency. However, these systems may exhibit lower long-term mechanical stability compared to permanently crosslinked networks, and their performance is sensitive to local biochemical conditions such as pH, enzyme activity, and substrate availability. As a result, enzymatic and dynamic crosslinking strategies are often integrated with physical or chemical crosslinking approaches to form hybrid networks that combine robustness with adaptability. Such synergistic designs are increasingly employed to meet the complex and evolving demands of regenerative medicine. The major crosslinking types, their underlying mechanisms, and their functional characteristics are summarized in Table 2

**Table 2:** Classification of crosslinking strategies in polysaccharide-based biomaterials

Crosslinking type	Mechanism	Key reactions	Typical polysaccharides	Advantages	Limitations	Biomedical applications	References
Physical crosslinking	Formation of networks through reversible non-covalent interactions resulting in supramolecular assembly	Hydrogen bonding, ionic interactions, electrostatic complexation, hydrophobic association, and chain entanglement	Alginate, cellulose, pectin, carrageenan, dextran, gellan gum, chitosan complexes	Mild processing conditions, high cytocompatibility, injectability, self-healing ability, and stimulus responsiveness	Lower mechanical strength and limited long-term structural stability; sensitive to environmental conditions such as ionic strength or dilution	Cell encapsulation, injectable hydrogels, wound healing matrices, and drug delivery systems	[134]
Chemical crosslinking	Permanent network formation through covalent bonding between functional groups on polysaccharide chains or chemically modified derivatives	Schiff-base reactions, carbodiimide-mediated amide bond formation, natural crosslinkers such as genipin, and photoinitiated polymerization	Hyaluronic acid, chitosan, alginate derivatives, oxidized polysaccharides, methacrylated polysaccharides	High mechanical strength, enhanced structural stability, controlled degradation, and long-term durability under physiological conditions	Potential cytotoxicity of reagents, requirement for reaction optimization, and possible restrictions on cell infiltration if crosslink density is excessive	Load-bearing scaffolds, long-term implants, sustained drug delivery platforms, and 3D bioprinted constructs	[135]
Enzymatic and dynamic crosslinking	Bioinspired network formation through enzyme-catalyzed reactions or reversible covalent and supramolecular bonds allowing adaptive remodeling	HRP-mediated oxidative coupling, transglutaminase catalysis, reversible Schiff-base bonds, boronate ester formation, and host–guest interactions	Hyaluronic acid derivatives, dextran, alginate derivatives, functionalized polysaccharide composites	High specificity, excellent cytocompatibility, controllable gelation kinetics, self-healing behavior, and dynamic stress relaxation	Lower long-term mechanical stability compared to permanent covalent networks; performance influenced by enzyme activity and biochemical environment	Injectable hydrogels, cell-laden scaffolds, stimuli-responsive biomaterials, and minimally invasive regenerative therapies	[136]

#### Design considerations for tunable network properties

The selection and integration of gelation and crosslinking mechanisms play a decisive role in defining the mechanical, structural, and biological performance of polysaccharide-based networks. Rather than relying on a single crosslinking strategy, effective scaffold design requires a holistic consideration of how physical, chemical, and enzymatic interactions collectively govern network architecture, stability, and dynamic behavior. These design considerations are particularly important in regenerative medicine, where scaffolds must simultaneously support mechanical loads, permit cellular remodeling, and respond adaptively to the evolving biological environment.

One of the primary design parameters is crosslinking density, which directly influences stiffness, elasticity, porosity, and degradation rate. Increasing crosslinking density generally enhances mechanica, strength and reduces swelling but may restrict cell migration and nutrient diffusion. Conversely, loosely crosslinked networks exhibit higher permeability and dynamic remodeling capacity but may lack sufficient mechanical integrity. Achieving an optimal balance requires careful tuning of polymer concentration, degree of functionalization, and crosslinking conditions, with consideration of tissue-specific mechanical requirements [97, 99]. Network homogeneity and spatial distribution of crosslinks are additional critical factors. Physical crosslinking often produces heterogeneous and reversible networks with localized domains of association, whereas chemical crosslinking tends to generate more uniform and permanent structures. Enzymatic and dynamic crosslinking systems introduce spatial and temporal control, enabling gradual network formation and stress relaxation. Combining these approaches allows the creation of hybrid networks that integrate the robustness of covalent bonds with the adaptability of reversible interactions [137, 138]. Such hybrid designs are particularly effective for mimicking the viscoelastic behavior of native extracellular matrices.

Degradation behavior must also be carefully coordinated with the crosslinking strategy. Chemically crosslinked networks typically degrade more slowly due to stable covalent bonds, while physically and dynamically crosslinked systems exhibit faster and more responsive degradation profiles. Enzymatic susceptibility can be tuned by modifying functional group accessibility or incorporating cleavable linkages, enabling degradation rates to be synchronized with tissue regeneration processes [49]. Failure to align degradation kinetics with biological remodeling can lead to premature scaffold collapse or prolonged foreign material presence.

Another key consideration is cytocompatibility during and after gelation. Physical and enzymatic crosslinking methods are generally preferred for cell-laden systems due to their mild reaction conditions and minimal use of reactive chemicals. In contrast, chemically crosslinked systems require careful control of reagent concentration, reaction time, and purification to avoid cytotoxic effects. The intended mode of scaffold deployment, whether injectable, in situ forming, or pre-fabricated, further influences crosslinking strategy selection and network design. Finally, network dynamics such as stress relaxation, self-healing, and responsiveness to environmental stimuli are increasingly recognized as critical determinants of biological performance. Dynamic networks formed through reversible bonds or supramolecular interactions allow cells to remodel their microenvironment, facilitating spreading, migration, and matrix deposition. Incorporation of such dynamic features can significantly enhance tissue integration and long-term functionality compared to static, permanently crosslinked systems [11, 139].

Collectively, these design considerations highlight that gelation, and crosslinking mechanisms should not be selected in isolation but rather as part of an integrated materials design strategy. By judiciously combining physical, chemical, and enzymatic approaches, polysaccharide-based networks can be engineered with tunable mechanical properties, controlled degradation, and adaptive biological behavior. These principles establish a mechanistic foundation for the fabrication and biofabrication strategies discussed in the following section, where material networks are translated into structured scaffolds.

## Fabrication and biofabrication of polysaccharide-based scaffolds

Fabrication and biofabrication strategies translate chemically engineered polysaccharide networks into 3D scaffolds with defined architecture, porosity, and mechanical performance. While gelation and crosslinking determine molecular-level network properties, fabrication methods govern macroscopic features such as pore size distribution, interconnectivity, surface morphology, and overall scaffold geometry. These structural attributes play a decisive role in cell infiltration, nutrient diffusion, vascularization, and tissue integration. Consequently, fabrication techniques must be selected in accordance with both material properties and intended biological function. Conventional scaffold fabrication methods remain widely used due to their simplicity, scalability, and compatibility with a broad range of polysaccharides. Techniques such as freeze-drying, cryogelation, solvent casting, and particulate leaching allow the generation of highly porous scaffolds without the need for complex equipment or harsh processing conditions. These approaches are often employed as baseline fabrication strategies or combined with advanced methods to create hierarchical structures.

### Conventional scaffold fabrication techniques

#### Freeze-drying and cryogelation

Freeze-drying and cryogelation are among the most extensively applied techniques for fabricating porous polysaccharide-based scaffolds. Both methods rely on ice crystal formation as a temporary porogen, enabling the creation of interconnected macroporous structures upon removal of the frozen solvent. These approaches are particularly attractive for polysaccharide systems because they allow aqueous processing, preserve bioactivity, and generate highly porous matrices suitable for tissue regeneration. Freeze-drying (lyophilization) involves freezing a polysaccharide solution or hydrogel followed by sublimation of the solvent under reduced pressure. During freezing, ice crystals nucleate and grow, expelling polymer chains into the interstitial regions; subsequent sublimation leaves behind a porous scaffold whose pore size, morphology, and interconnectivity are governed by freezing conditions such as cooling rate, freezing temperature, polymer concentration, and solvent composition [10, 140]. Rapid freezing typically produces smaller, more homogeneous pores, whereas slow freezing yields larger and more anisotropic pore structures. Recent advances in freeze-drying techniques such as directional freeze-drying, freeze-gelation, and controlled sublimation enable more precise control over pore architecture, mechanical anisotropy, and scaffold performance [104]. These innovations have improved outcomes in nerve regeneration, osteochondral repair, and drug-eluting implants. Despite its advantages, freeze-drying remains energy-intensive, time-consuming, and can yield brittle scaffolds that require post-processing; solvent residue and cryoprotectant compatibility remain important considerations for in vivo use [141]. For example, a new method combining freeze-drying and cross-linking was developed to produce biocompatible, porous polysaccharide scaffolds without using organic solvents or porogens. Pullulan and dextran were successfully crosslinked with sodium trimetaphosphate during freeze-drying, and pore size and porosity were controlled by adjusting freeze-drying pressure. This approach enables the fabrication of tunable 3D scaffolds suitable for tissue engineering and biomedical applications [142].

**Fig. 2:**
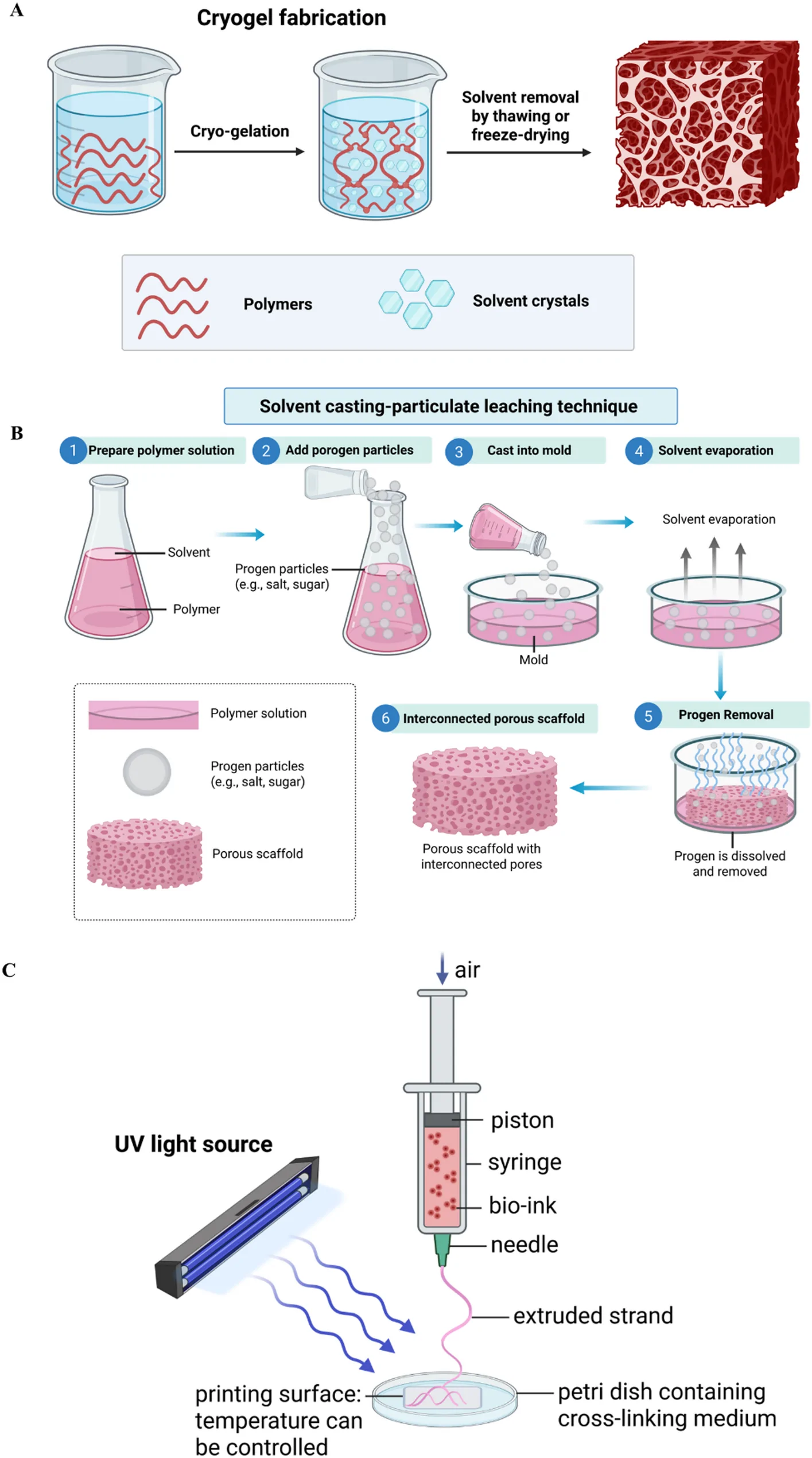
Fabrication strategies for porous scaffolds and biofabrication. (A) Schematic illustration of cryogel fabrication, adapted from Carriero et al. [144]. (B) Schematic illustration of the solvent casting–particulate leaching technique, adapted from Sampath et al. [145]. (C) Schematic illustration of extrusion-based bioprinting, adapted from You et al. [146]

Cryogelation differs from conventional freeze-drying in that crosslinking occurs while the system is partially frozen. As illustrated in Fig. 2a, the process begins with dissolution of the matrix components, followed by solvent freezing that generates solvent crystals acting as porogens while polymer chains concentrate in the unfrozen microphase. Crosslinking reactions proceed within these polymer-rich regions, forming a stabilized network around the solvent crystals. Subsequent thawing or freeze-drying removes the frozen solvent, leaving an interconnected macroporous cryogel structure with thick pore walls and enhanced mechanical robustness. This architecture typically yields cryogels that exhibit elastic behavior, high compressibility, and shape recovery, making them particularly suitable for injectable or minimally invasive scaffold delivery [143]. Although freeze-dried scaffolds may suffer from brittleness and cryogels may provide lower structural resolution than some advanced biofabrication methods, both techniques remain highly valuable due to their reproducibility, scalability, and compatibility with sensitive biological components such as proteins and growth factors.

#### Solvent casting and particulate leaching

Solvent casting combined with particulate leaching is a widely used fabrication strategy for generating porous polysaccharide-based scaffolds with controlled pore size and high interconnectivity. This technique relies on the incorporation of a removable porogen within a polymer solution, followed by solvent evaporation to form a solid matrix and subsequent leaching of the porogen to create void spaces [147]. Owing to its conceptual simplicity, low equipment requirements, and compatibility with a wide range of polysaccharides, solvent casting–particulate leaching remains a foundational method for scaffold fabrication in tissue engineering.

As illustrated in Fig. 2b, the process begins with preparation of a homogeneous polymer solution, into which solid porogen particles are uniformly dispersed. The mixture is then cast into a mold of the desired geometry, where solvent evaporation causes the polymer chains to consolidate and form a continuous matrix surrounding the porogen particles. After solidification, the construct is immersed in an appropriate solvent to dissolve and remove the porogen, leaving behind a 3D interconnected porous scaffold whose pore size, distribution, and interconnectivity are dictated primarily by the size, shape, packing density, and concentration of the porogen. This process effectively decouples pore architecture from polymer chemistry, allowing independent tuning of structural parameters [145].

For polysaccharide systems, solvent casting is typically conducted using aqueous or mildly acidic solutions to preserve biocompatibility and prevent degradation of sensitive functional groups. Polysaccharides such as chitosan, alginate, cellulose derivatives, and dextran have been successfully processed using this method, often in combination with post-fabrication crosslinking to enhance mechanical stability [10, 148]. The resulting scaffolds commonly exhibit well-defined macroporosity and interconnected networks that facilitate cell infiltration and mass transport. Control over scaffold architecture is achieved through several adjustable parameters. Increasing porogen content enhances overall porosity but may compromise mechanical integrity, while porogen size directly influences pore diameter. Solvent evaporation rate affects polymer chain packing and surface morphology, with slow evaporation generally producing more uniform structures. However, because pore interconnectivity depends on porogen contact during casting, achieving fully interconnected networks requires careful optimization of porogen loading and packing density. Sola et al. [149] used the solvent-casting particulate leaching (SCPL) method to create highly porous 3D scaffolds from poly (methyl methacrylate) [PMMA] and polyurethane using salt as a porogen. By adjusting the polymer-to-salt ratio and salt grain size, pore structure and mechanical properties were controlled. The resulting scaffolds showed high interconnected porosity and supported stromal cell growth that maintained their protective effect on leukemic cells. These 3D platforms can better mimic the bone marrow niche and help evaluate new anticancer therapies.

Despite its versatility, solvent casting–particulate leaching presents inherent limitations. Residual porogen or solvent traces may persist if leaching is incomplete, necessitating thorough washing steps. Additionally, scaffold thickness is constrained by diffusion limits during solvent evaporation and porogen removal, which can result in heterogeneous pore structures in thicker constructs. Mechanical properties are often inferior to those obtained via cryogelation or covalent crosslinking unless reinforced through chemical modification or composite formulation [150]. While increasingly complemented by advanced biofabrication methods, solvent casting–particulate leaching remains a valuable and accessible approach for producing porous polysaccharide scaffolds and for benchmarking structure–function relationships in scaffold design.

### Fiber- and particle-based fabrication

Fiber- and particle-based fabrication techniques enable the construction of polysaccharide scaffolds with high surface area, anisotropic architecture, and finely controlled micro- to nanoscale features. Unlike bulk fabrication methods, these approaches generate discrete structural units, fibers or particles that can be assembled into mats, meshes, or composite scaffolds. Such architectures more closely resemble the fibrous organization of native extracellular matrices and offer enhanced control over cell–material interactions, mass transport, and mechanical anisotropy. Among these approaches, electrospinning and wet spinning are the most widely adopted techniques for producing continuous polysaccharide fibers. While both methods rely on polymer solution processing, they differ fundamentally in their driving forces, fiber dimensions, and scalability, making them complementary tools for scaffold fabrication.

#### Electrospinning and wet spinning

Electrospinning is a versatile fabrication technique that uses a high-voltage electric field to generate continuous polymer fibers with diameters ranging from several micrometers down to the nanometer scale. In a typical electrospinning setup, a charged polysaccharide solution is extruded through a nozzle, forming a Taylor cone and a fine jet that undergoes elongation and solvent evaporation before depositing as a nonwoven fibrous mat [151]. The resulting electrospun scaffolds exhibit high surface area-to-volume ratios, interconnected porosity, and fiber morphologies that closely mimic native ECM fibrillar networks.

Polysaccharide electrospinning presents specific challenges due to their polyelectrolyte nature, high surface tension, and limited solubility in volatile organic solvents. As a result, electrospinning often requires chemical modification, blending with synthetic polymers, or the use of specialized solvent systems to achieve stable jet formation [152]. Commonly, electrospun polysaccharides include chitosan, alginate, cellulose derivatives, hyaluronic acid, and dextran, either alone or in composite formulations. For example, Tungprapa et al. [153] reported electrospun nanofibers of cellulose acetate loaded with four types of non-steroidal anti-inflammatory drugs (NSAIDs) [naproxen, indomethacin, ibuprofen, and sulindac] that are utilized in the symptomatic management of painful and inflammatory conditions. Also, Snetkov et al. [154] fabricated electrospun nanofibrous textiles from native hyaluronic acid alone, without using additional carrier polymers or modifiers, and successfully incorporated curcumin and usnic acid. They suggested that these pure hyaluronic acid textiles could serve as promising materials for wound dressings and drug delivery scaffolds. Fiber diameter, alignment, and scaffold thickness can be precisely tuned by adjusting solution properties such as viscosity, conductivity, and polymer concentration, as well as process parameters including applied voltage, flow rate, and collector geometry.

Wet spinning provides an alternative fiber fabrication route that is particularly well suited for polysaccharides with limited electrospinnability. In this process, a polysaccharide solution is extruded into a coagulation bath containing a non-solvent or crosslinking agent, inducing fiber solidification through phase inversion or ionic crosslinking [155]. Wet-spun fibers typically possess larger diameters than electrospun fibers, ranging from tens to hundreds of micrometers, and exhibit higher mechanical strength and uniformity. Polysaccharides such as alginate, chitosan, cellulose, and gellan gum are frequently processed via wet spinning due to their ability to undergo rapid ionic or pH-induced gelation. The study of Cui et al. [156] developed a simple wet-spinning method to produce edible composite fibers from sodium alginate and soybean protein isolate to better mimic the texture of animal muscle in meat alternatives.

The distinct characteristics of electrospun and wet-spun fibers enable different scaffold design strategies. Electrospun mats provide nanoscale topographical cues and high surface area but often suffer from limited pore size and restricted cell infiltration unless modified through multilayering or post-processing. Wet-spun fibers, by contrast, can be woven, braided, or assembled into 3D constructs with well-defined macroporosity and load-bearing capability [157]. Combining electrospinning and wet spinning within hybrid scaffolds allows integration of nanoscale bioactivity with macroscale mechanical support. Together, electrospinning and wet spinning represent powerful and complementary approaches for engineering fibrous polysaccharide scaffolds with tailored structural and functional properties.

#### Emulsification and micro/nanoparticle encapsulation

Emulsification and micro/nanoparticle encapsulation are discussed here specifically as fabrication tools for generating particulate building blocks that define scaffold microarchitecture, rather than as drug or therapeutic delivery systems [158]. These techniques enable the production of discrete polysaccharide particles with controlled size, morphology, and surface characteristics that can function as independent structural units or be integrated into bulk scaffolds to introduce hierarchical organization, controlled porosity, and localized functional domains. Compared to fiber-based methods, particle fabrication offers greater flexibility in tuning microscale architecture and enables modular scaffold design through particle assembly or embedding within continuous matrices [159, 160].

Emulsification-based fabrication relies on the formation of droplets from a polysaccharide-containing phase dispersed within an immiscible continuous phase, followed by solidification through physical or chemical crosslinking. Oil-in-water and water-in-oil emulsions are commonly employed, depending on polysaccharide solubility and crosslinking strategy. Droplet size, and consequently particle size, is governed by parameters such as emulsifier type and concentration, stirring speed, viscosity ratio between phases, and temperature [105]. Subsequent crosslinking often achieved through ionic interactions, chemical reactions, or thermal gelation, stabilizes the droplets into solid or gelled micro- or nanoparticles. Ionic gelation is particularly well suited for anionic polysaccharides such as alginate and pectin, where divalent cations induce rapid particle formation at the droplet interface. Chitosan-based particles are frequently fabricated through ionic complexation with multivalent anions or through pH-induced precipitation. These approaches proceed under mild aqueous conditions and preserve polysaccharide bioactivity, making them compatible with sensitive biological components [161]. Particle size can range from several micrometers to the nanoscale, depending on emulsification energy and formulation parameters.

Micro- and nanoparticle fabrication strategies are often combined with additional processing steps to improve uniformity and scalability. Techniques such as double emulsion formation, solvent evaporation, and spray-based emulsification enable tighter control over particle size distribution and structural consistency. Polysaccharide particles produced through these methods exhibit high surface area-to-volume ratios and tunable degradation behavior, which can be exploited to modulate scaffold microarchitecture when embedded within larger constructs [10, 162]. For example, de La Taille et al. [163] developed stable, biocompatible polysaccharide hydrogel nanoparticles (~ 315 nm) using a microemulsion method and functionalized them with fucoidan to target P-selectin on thrombi. The nanoparticles could be loaded with recombinant tissue-type plasminogen activator (rtPA) or deoxyribonuclease I (DNase I) and showed effective, targeted clot breakdown, including synergistic action against neutrophil extracellular traps (NET)-rich thrombi. In vivo, rtPA-loaded nanoparticles achieved improved thrombolysis at reduced doses without causing secondary embolism, highlighting their promise as safe and efficient thrombolytic drug delivery systems. Microfluidic emulsification has further advanced particle fabrication by enabling highly uniform droplet generation with narrow size distributions. In these systems, controlled fluid flow through microchannels produces monodisperse polysaccharide droplets that can be crosslinked on-chip or downstream [164]. Microfluidic approaches offer exceptional reproducibility and precision, although throughput limitations currently restrict large-scale production. Nevertheless, they provide valuable platforms for systematically studying structure–function relationships in particulate polysaccharide scaffolds.

When incorporated into bulk scaffolds, polysaccharide micro- and nanoparticles can serve multiple structural roles, including porogens, reinforcing fillers, or localized architectural domains. Particle incorporation increases surface roughness, modulates pore interconnectivity, and introduces a multiscale architecture that enhances mass transport and mechanical performance [165]. However, excessive particle loading or poor interfacial integration may compromise scaffold integrity, underscoring the need for careful design of particle–matrix interactions. Together, these approaches expand the fabrication repertoire for polysaccharide scaffolds and provide modular building blocks for advanced biofabrication strategies discussed in the following section.

### Advanced biofabrication approaches

Advanced biofabrication techniques enable precise spatial control over scaffold architecture, composition, and biological functionality, surpassing the limitations of conventional fabrication methods. These approaches integrate material processing with digital design and automated manufacturing, allowing the construction of complex, patient-specific scaffolds with hierarchical organization. For polysaccharide-based biomaterials, advanced biofabrication leverages their tunable rheological properties, mild gelation behavior, and cytocompatibility to enable controlled deposition and in situ solidification. Among these techniques, 3D bioprinting and extrusion-based printing have emerged as the most widely adopted strategies.

#### Three-dimensional bioprinting and extrusion-based printing

Three-dimensional bioprinting is an additive manufacturing technique in which bioinks are deposited layer by layer to generate predefined 3D constructs with controlled geometry and internal architecture. Extrusion-based printing, the most commonly used bioprinting modality for polysaccharide systems, relies on pneumatic or mechanical forces to extrude continuous filaments through a nozzle, followed by stabilization through physical, chemical, or enzymatic crosslinking [166]. This approach offers compatibility with a wide range of viscosities, materials, and cell densities, making it particularly suitable for polysaccharide-based bioinks. The study of Nikolaou et al. [167] evaluated starch-based gel formulations containing different proteins and hydrocolloids for extrusion-based 3D food printing.

As illustrated in Fig. 2C, extrusion-based bioprinting involves loading the polysaccharide bioink into a syringe or cartridge, where controlled pneumatic pressure or mechanical piston-driven force pushes the material through a fine needle to produce a continuous filament that is deposited onto a printing surface following a predefined pattern. During or immediately after extrusion, crosslinking can be initiated using different mechanisms depending on the material system. For example, photo-crosslinkable formulations may be exposed to UV or visible light to induce rapid polymerization, whereas ionic or thermal crosslinking can occur upon contact with a crosslinking medium or temperature-controlled substrate. These coordinated steps enable precise layer-by-layer construction of 3D scaffolds while maintaining filament shape fidelity and structural stability. Polysaccharides are highly attractive candidates for bioink formulation due to their shear-thinning behavior, rapid gelation under mild conditions, and inherent biocompatibility. Materials such as alginate, hyaluronic acid, chitosan, cellulose derivatives, and polysaccharide-based composite bioinks have been extensively explored as printable matrices [168, 169]. Printability is governed by rheological properties, including viscosity, yield stress, and thixotropy, which must be carefully balanced to ensure smooth extrusion, shape fidelity, and post-printing structural stability. These properties are commonly tuned through polymer concentration, molecular weight, degree of functionalization, and incorporation of rheology modifiers.

Extrusion-based printing enables precise control over filament diameter, pore geometry, and scaffold anisotropy through adjustment of printing parameters such as nozzle diameter, extrusion pressure, printing speed, and layer height. Post-printing crosslinking strategies, including ionic gelation, photopolymerization, or enzymatic reactions, are often employed to reinforce printed constructs and prevent deformation. Hybrid crosslinking approaches are frequently used to combine immediate shape retention with long-term mechanical stability, particularly in cell-laden systems [170]. Despite its versatility, extrusion-based bioprinting presents several challenges when applied to polysaccharide bioinks. Limited mechanical strength, diffusion-driven swelling, and delayed crosslinking can compromise print fidelity and dimensional accuracy. Additionally, nozzle-induced shear stress may affect cell viability if bioink rheology is not properly optimized. Strategies such as composite bioink formulation, sacrificial support materials, and dynamic crosslinking systems have been developed to address these limitations and expand the printable design space. One of the key advantages of 3D bioprinting is its capacity for spatially controlled material and cell placement, allowing multimaterial deposition and the creation of heterogeneous scaffolds with gradients in stiffness, porosity, or biochemical composition. Together, these strategies establish a versatile platform for translating chemically and physically tuned polysaccharide systems into programmable, 3D scaffolds.

#### Microfluidic and microfabrication strategies

Microfluidic and microfabrication strategies enable precise control over scaffold architecture and component size at the micrometer and submicrometer scales, offering capabilities that complement extrusion-based bioprinting. These approaches rely on engineered microchannels, patterned templates, or lithographic techniques to manipulate polysaccharide materials with high spatial resolution, enabling reproducible fabrication of microstructured scaffolds, fibers, and particles [171]. For polysaccharide-based systems, microfluidic and microfabrication techniques are particularly attractive due to their compatibility with aqueous processing, mild gelation mechanisms, and ability to generate uniform and well-defined structures.

Microfluidic fabrication exploits laminar flow behavior within microchannels to precisely control fluid interfaces, mixing, and droplet formation. In polysaccharide systems, this capability has been widely used to generate monodisperse microgels, fibers, and capsules through controlled emulsification, ionic crosslinking, or enzymatic gelation [172]. By adjusting flow rates, channel geometry, and crosslinking conditions, microfluidic platforms enable fine control over feature size, morphology, and internal structure, often achieving levels of uniformity that are difficult to obtain with bulk emulsification methods. Microfluidic fiber fabrication represents an extension of wet spinning at the microscale, where coaxial or multilayer flow configurations are used to generate continuous fibers with controlled diameter and composition. Polysaccharides such as alginate, chitosan, and cellulose derivatives readily undergo ionic or pH-triggered gelation within microchannels, enabling fabrication of fibers with tailored cross-sections, core–shell architectures, or compositional gradients [173]. These features allow precise tuning of mechanical properties and facilitate the integration of multiple material phases within a single construct. The work of Iijima et al. [174] developed flexible, water-insoluble hollow and solid fibers from natural polysaccharides by forming chondroitin sulfate–chitosan polyion complexes using a coaxial microfluidic device.

Beyond microfluidics, microfabrication techniques such as soft lithography, micromolding, and photopatterning enable the creation of polysaccharide scaffolds with predefined geometries and surface features. In micromolding approaches, polysaccharide solutions or hydrogels are cast into patterned molds, producing scaffolds with well-defined pore shapes, channels, or surface topographies. Photopatterning strategies, often used in conjunction with photo-crosslinkable polysaccharides, allow spatially selective gelation and pattern formation with high resolution [175]. These techniques are particularly useful for generating microarchitectures that guide cell alignment, migration, or tissue organization, although their biological implications are discussed in later sections.

A key advantage of microfluidic and microfabrication strategies is their reproducibility and precision, which facilitate systematic investigation of structure–function relationships. However, these methods also face practical limitations, including lower throughput, increased fabrication complexity, and challenges in scaling to clinically relevant dimensions. As a result, microfabrication approaches are often integrated with extrusion-based printing or bulk fabrication techniques to create multiscale scaffolds that combine microscale precision with macroscale form. Together, these strategies expand the design space for polysaccharide-based scaffolds and provide powerful tools for engineering complex architectures with controlled microstructural features.

### Structure–function relationships in fabricated scaffolds

The functional performance of polysaccharide-based scaffolds is governed not only by material composition and crosslinking chemistry but also by the hierarchical architecture established during fabrication. Structural features introduced at the macro-, micro-, and nanoscale directly influence mechanical behavior, mass transport, cell–material interactions, and long-term tissue integration. Understanding these structure–function relationships is essential for rational scaffold design and for selecting fabrication strategies that align material properties with biological requirements.

At the macroscale, overall scaffold geometry and pore architecture determine bulk mechanical performance and fluid transport. Fabrication techniques such as freeze-drying, cryogelation, and solvent casting produce highly porous structures with interconnected macropores that facilitate nutrient diffusion and waste removal. However, pore size and interconnectivity must be carefully balanced, as excessive porosity can compromise mechanical integrity, while insufficient porosity restricts infiltration and mass transport. Control over freezing rate, porogen size, and polymer concentration enables tuning of macroporous architecture to meet specific structural demands [176, 177].

At the microscale, scaffold architecture influences stress distribution, deformation behavior, and local transport phenomena. Fiber-based fabrication methods, including electrospinning and wet spinning, generate anisotropic structures with aligned or woven fiber networks that impart direction-dependent mechanical properties. Electrospun nanofibrous mats provide high surface area and nanoscale topographical cues, while wet-spun fibers offer greater load-bearing capacity and macroporous assembly potential. Similarly, incorporation of micro- and nanoparticles fabricated through emulsification or microfluidics introduces controlled heterogeneity, modulating pore interconnectivity, stiffness gradients, and local microenvironments within composite scaffolds [152, 178].

Nanoscale features play a critical role in governing interfacial phenomena, including protein adsorption and initial cell attachment. Fabrication-induced surface roughness, fiber diameter, and particle size influence the organization of adsorbed protein layers, which in turn regulate receptor engagement and downstream cellular responses. Electrospinning and microfabrication techniques are particularly effective at introducing nanoscale features that approximate the fibrillar architecture of native extracellular matrices, enhancing scaffold biointegration without altering bulk material chemistry [151, 175].

Fabrication strategy also dictates mechanical anisotropy and viscoelastic behavior, which are increasingly recognized as important determinants of biological performance. Extrusion-based bioprinting and microfabrication allow spatial control over filament orientation, layer stacking, and material composition, enabling the creation of scaffolds with region-specific stiffness and deformation profiles. Such structural programmability is difficult to achieve with conventional fabrication methods and represents a major advantage of advanced biofabrication approaches [166].

Importantly, structure–function relationships are inherently coupled to crosslinking and material chemistry. Identical polysaccharide formulations can exhibit markedly different functional behavior depending on fabrication route, as differences in pore architecture, fiber alignment, and surface topology alter mechanical load transfer and biological interactions. Consequently, fabrication should be viewed not as a downstream processing step but as an integral design variable that operates alongside chemical modification and gelation strategy. Collectively, these relationships underscore the necessity of aligning fabrication method, scaffold architecture, and intended function within a unified design framework. By selecting appropriate fabrication strategies and tuning structural features across multiple length scales, polysaccharide-based scaffolds can be engineered to achieve predictable mechanical performance, efficient mass transport, and favorable biological interfaces. These principles provide a structural foundation for the biofunctionalization strategies and application-specific designs discussed in subsequent sections.

## Immune-instructive and biofunctional design strategies

Beyond providing structural support, polysaccharide-based scaffolds actively regulate cellular behavior through biochemical, mechanical, and immunological cues. Biofunctionalization strategies enable these materials to transition from passive matrices into instructive microenvironments that guide cell fate, immune response, and tissue remodeling. By leveraging the context-dependent biofunctionality of polysaccharides and integrating targeted functional motifs, scaffolds can be engineered to modulate inflammation, enhance regeneration, and promote long-term tissue integration.

### Cell–material interactions and immune modulation

Cell–material interactions are critical determinants of scaffold performance in regenerative medicine because they influence cell adhesion, migration, proliferation, differentiation, immune activation, and subsequent tissue remodeling. Polysaccharide-based biomaterials are particularly relevant in this context because many share structural or chemical features with ECM components, especially glycosaminoglycans, which participate in cell signaling and tissue homeostasis [179]. Through their intrinsic molecular characteristics and engineered modifications, polysaccharide scaffolds can influence both cellular behavior and the local immune microenvironment. At the material interface, cell responses can arise through indirect physicochemical mechanisms. Surface charge, hydrophilicity, stiffness, roughness, and topography influence the adsorption, orientation, and conformation of proteins from the surrounding biological environment. Anionic polysaccharides, including alginate, hyaluronic acid, heparin, and chondroitin sulfate, can therefore affect cell adhesion indirectly by altering the composition and organization of the adsorbed protein layer [180]. Because many polysaccharides lack intrinsic integrin-binding sequences, they are frequently functionalized with cell-adhesive peptides, growth-factor-binding domains, or ECM-mimetic ligands. For example, incorporation of arginine–glycine–aspartic acid (RGD) motifs into polysaccharide-based scaffolds can enhance integrin-mediated attachment and cell–matrix signaling [181].

Importantly, these indirect interfacial effects should be distinguished from direct stereospecific receptor-mediated recognition. Physicochemical properties such as hydrophilicity, surface chemistry, and topography primarily influence immune responses by modifying protein adsorption and the resulting interfacial protein corona [182]. By contrast, interactions such as hyaluronic acid binding to CD44 or β-glucan recognition by Dectin-1 depend on defined molecular structures and ligand–receptor complementarity [183]. Thus, immune responses to polysaccharide biomaterials may arise through direct carbohydrate–receptor signaling, indirect protein-corona-mediated pathways, or a combination of both mechanisms, and these processes should not be conflated.

Direct polysaccharide recognition is strongly influenced by glycan structure. Defined carbohydrate motifs can be recognized by C-type lectin receptors, including Dectin-family receptors, as well as by galectins and Siglecs. Receptor engagement depends on parameters such as monosaccharide stereochemistry, glycosidic linkage, branching, sulfation, molecular weight, chain conformation, and 3D presentation. Lee et al. [58] demonstrated that structural differences among yeast-derived β-glucan-containing polysaccharides can lead to distinct Dectin-1-dependent immune responses, indicating that glycan architecture is directly linked to biological function. Dectin-1 A recognition of β-(1,3;1,6)-glucans is also influenced by solution conformation and higher-order glucan organization, which affect receptor clustering and downstream signaling. Similarly, Choi et al. [59] emphasized the importance of specific carbohydrate motifs and stereochemical presentation in carbohydrate–protein recognition. Accordingly, the immunomodulatory behavior of polysaccharide scaffolds is more appropriately interpreted within a structure–recognition–signaling framework than as a generalized consequence of surface charge or natural-polymer origin. Protein-corona-mediated effects represent a mechanistically distinct pathway. Surface chemistry and wettability can alter not only the quantity but also the identity and conformation of adsorbed proteins, which subsequently influence macrophage activation and inflammatory signaling. Studies using nanoengineered surfaces have shown that changes in surface topography or wettability can modify protein-corona composition and subsequently alter macrophage inflammatory responses [184, 185]. These observations reinforce the need to distinguish indirect immune regulation caused by material-controlled protein adsorption from direct stereospecific recognition of polysaccharide ligands by receptors such as Dectin-1 and CD44.

Immune modulation has emerged as an important design objective for advanced polysaccharide scaffolds. Rather than merely minimizing inflammation, contemporary biomaterials increasingly aim to guide immune responses toward conditions that support repair. Functionalized polysaccharides can interact with pattern-recognition and adhesion receptors, including TLRs, Dectin-1, and CD44, and thereby influence macrophage activation, cytokine production, angiogenesis, matrix remodeling, and classification. Polysaccharide-based materials should therefore be described as biasing tissue repair [11, 14]. However, macrophage activation is now understood as a dynamic spectrum rather than a strict binary M1/M2 macrophage signaling toward inflammatory, resolving, or reparative profiles under defined experimental conditions, rather than as imposing fixed polarization states.

A critical qualification is required when immune responses are attributed to the intrinsic chemistry of polysaccharides. Residual endotoxin can activate innate immune pathways and induce cytokine release, thereby mimicking, amplifying, or masking polysaccharide-dependent effects. This concern is particularly important for chitosan and microbial polysaccharides, for which source, molecular weight, extraction, purification, and structural organization can vary substantially. Lee et al. [62] showed that chitosan-mediated immune responses depend strongly on physicochemical characteristics and interactions with pattern-recognition pathways, helping to explain apparently conflicting inflammatory and anti-inflammatory observations. Lee et al. [24] similarly highlighted the dependence of chitin/chitosan immune activity on molecular weight, formulation, and experimental conditions. Consequently, claims of M1-to-M2 polarization, anti-inflammatory activity, or cytokine modulation should not be attributed exclusively to polysaccharide chemistry unless material purity and endotoxin burden have been appropriately characterized. The sulfation pattern, molecular weight, and charge density of polysaccharides further influence immune and regenerative signaling. Highly sulfated polysaccharides such as heparin and fucoidan can bind growth factors and cytokines, altering their stability and local bioavailability within scaffolds [186]. Hyaluronic acid also exhibits molecular-weight-dependent immunological behavior, with fragments of different sizes producing distinct receptor-mediated and inflammatory responses [44]. These examples further emphasize that polysaccharide bioactivity depends on defined structural parameters rather than polymer identity alone. Representative polysaccharides, their principal receptor or interaction mechanisms, reported immunological effects, and regenerative applications are summarized in Table 3.

Scaffold architecture and mechanical properties provide an additional layer of immune regulation through mechanotransduction and cell–matrix interactions. Parameters such as pore size, fiber alignment, viscoelasticity, stiffness, and degradation kinetics can affect immune-cell infiltration, macrophage behavior, fibroblast activation, and vascular remodeling. Softer or dynamically remodeling polysaccharide networks have been associated in some systems with reduced fibrotic responses, whereas excessively stiff or poorly degradable matrices may promote prolonged foreign-body reactions [187]. Polysaccharide scaffolds also interact with dendritic cells, fibroblasts, endothelial cells, and other stromal populations, allowing material chemistry, architecture, and degradation to collectively influence angiogenesis and tissue repair [188]. Collectively, these observations support the concept of polysaccharides as immune-interactive and potentially immune-instructive biomaterials rather than inert scaffold components. Rational design therefore requires simultaneous consideration of molecular recognition, protein adsorption, material mechanics, degradation, purity, and biological context. Integrating these mechanisms provides a stronger basis for designing polysaccharide scaffolds that promote regenerative signaling while minimizing uncontrolled inflammatory or foreign-body responses.

**Table 3:** Immunomodulatory mechanisms and regenerative applications of polysaccharide-based biomaterials

Polysaccharide/material	Receptor/ interaction mechanism	Immunological effect	Regenerative application	References
Hyaluronic acid	CD44; RHAMM; molecular-weight-dependent receptor signaling	Modulation of inflammatory signaling, cell migration, and macrophage responses; effects depend strongly on molecular weight and fragmentation state	Skin repair, cartilage regeneration, neural applications	[189, 190]
Chitosan	TLR2/TLR4 and other pattern-recognition pathways; effects depend on molecular weight and formulation	Context-dependent inflammatory or resolving responses; IL-10-associated and reparative macrophage responses have been reported in selected systems	Bone and skin regeneration	[191]
β-Glucan	Dectin-1 and other C-type lectin receptors	Glycan-structure- and conformation-dependent regulation of phagocytosis, cytokine signaling, and macrophage activation	Wound healing; immune-regenerative applications	[192, 193]
Sulfated polysaccharides	Growth-factor/cytokine binding; receptor interactions dependent on sulfation pattern	Modulation of growth-factor stability, inflammatory signaling, and reparative immune responses	Bone and cardiovascular tissue engineering	[7, 186]
Modified alginate	Predominantly indirect ECM-mimetic and interfacial mechanisms; receptor effects depend on modification	Modulation of cell recruitment and local inflammatory responses; reparative macrophage bias reported in selected formulations	Cardiac repair; islet transplantation	[191]
Fucoidan	Selectins, growth-factor interactions, and pattern-recognition pathways	Modulation of inflammatory signaling, growth-factor activity, and angiogenic responses	Vascular and liver tissue regeneration	[194, 195]
Zwitterionic polysaccharide-based materials	Predominantly interfacial/protein-adsorption-mediated mechanisms	Reduced nonspecific protein adsorption and attenuated inflammatory/fibrotic responses in selected systems	Implant integration	[196]

CD44, cluster of differentiation 44; ECM, extracellular matrix; IL-10, interleukin-10; RHAMM, receptor for hyaluronan-mediated motility; TGF-β, transforming growth factor-beta; TLR2, Toll-like receptor 2; TLR4, Toll-like receptor 4

### Scaffold-mediated presentation of bioactive signals

A central advantage of polysaccharide-based scaffolds is their capacity to act as reservoirs and delivery platforms for bioactive molecules, enabling localized, sustained, and biologically relevant signaling within regenerative microenvironments. Unlike passive carriers, polysaccharide matrices can regulate the spatial and temporal presentation of therapeutic cues through a combination of physical entrapment, affinity binding, and stimulus-responsive release. This delivery capability complements their structural and immunomodulatory functions, allowing scaffolds to actively instruct cellular behavior during tissue repair and regeneration. Polysaccharide-mediated delivery strategies are particularly attractive because they rely on mild processing conditions, preserve biomolecule bioactivity, and can be readily integrated into hydrogel, fiber-based, and biofabricated constructs. Growth factors, cytokines, and small bioactive molecules represent the most extensively explored payloads and form the foundation of instructive scaffold design.

#### Growth factors, cytokines, and small molecules

Growth factors and cytokines play essential roles in regulating cell proliferation, differentiation, angiogenesis, and immune responses during tissue regeneration. Polysaccharide-based scaffolds provide a versatile platform for their delivery by mimicking the natural ECM, which sequesters and presents signaling molecules through electrostatic and affinity-based interactions. Polysaccharides containing charged functional groups, particularly carboxylated and sulfated derivatives, exhibit strong binding affinity for heparin-binding growth factors and cytokines, enabling sustained and localized signaling [46, 47].

Sulfated polysaccharides such as heparin, fucoidan, and sulfated chitosan are especially effective at stabilizing growth factors by protecting them from enzymatic degradation and prolonging their biological activity. By regulating binding strength through sulfation degree and spatial distribution, these materials can modulate release kinetics and bioavailability. Hyaluronic acid and alginate-based matrices, although less strongly binding, enable controlled diffusion-driven release when crosslinking density and pore architecture are carefully tuned [10]. Growth factor delivery from polysaccharide scaffolds can be achieved through several complementary mechanisms. Physical encapsulation within hydrogels or porous matrices allows diffusion-controlled release, which is primarily governed by mesh size, swelling behavior, and degradation rate. Affinity-based delivery leverages specific interactions between polysaccharide functional groups and growth factor domains, enabling prolonged retention and reduced burst release. Covalent immobilization strategies, while less commonly used due to concerns about activity loss, allow precise spatial patterning of signaling cues when carefully designed [97].

In addition to growth factors, polysaccharide scaffolds are increasingly used to deliver cytokines and small immunoregulatory molecules that shape the inflammatory microenvironment. Controlled cytokine presentation enables scaffolds to attenuate excessive inflammation while promoting regenerative immune phenotypes. Small molecules, including anti-inflammatory agents, angiogenic modulators, and osteoinductive compounds, benefit from polysaccharide-based delivery due to enhanced local retention and reduced systemic exposure. Release profiles can be tuned through polymer composition, crosslinking mechanism, and incorporation of micro- or nanoparticulate carriers within the scaffold matrix [10, 162].

An important design consideration is the coupling between scaffold degradation and bioactive molecule release. As polysaccharide matrices undergo enzymatic or hydrolytic degradation, encapsulated molecules are progressively liberated, allowing release kinetics to be synchronized with tissue regeneration processes. This coupling enables dynamic signaling environments that evolve, more closely reflecting natural healing cascades. Together, these delivery strategies position polysaccharide scaffolds as multifunctional platforms capable of orchestrating complex biological signaling through controlled presentation of soluble cues.

#### Exosomes, nucleic acids, and nanotherapeutics

Beyond conventional growth factors and small molecules, polysaccharide-based scaffolds have emerged as versatile platforms for the delivery of advanced biological payloads, including extracellular vesicles, nucleic acids, and nanotherapeutics [197]. These payloads enable more sophisticated modes of cellular instruction by modulating gene expression, intercellular communication, and intracellular signaling pathways. In these systems, polysaccharide scaffolds function as stabilizing and spatially organizing matrices rather than as bioactive agents themselves, with biological effects arising from controlled presentation of extracellular vesicle cargo. The physicochemical properties of polysaccharides, including charge tunability, degradability, and affinity for biomolecules, provide a favorable environment for stabilizing and presenting these complex therapeutic agents.

Exosomes and other extracellular vesicles represent a particularly promising class of bioactive cargo due to their role in mediating cell–cell communication and tissue regeneration. Derived from stem cells, immune cells, or tissue-specific sources, exosomes carry proteins, lipids, and nucleic acids that collectively influence angiogenesis, immunomodulation, and matrix remodeling [198]. However, their clinical translation is limited by rapid clearance, poor retention at target sites, and susceptibility to degradation. Polysaccharide-based scaffolds address these challenges by serving as localized reservoirs that protect exosomes from premature degradation and enable sustained release within the regenerative microenvironment [9, 15]. Electrostatic interactions, physical entrapment within hydrogel networks, and affinity-based binding to sulfated or charged polysaccharides contribute to enhanced exosome retention and bioactivity. Lin et al. [199] developed a dual-dynamic covalent hydrogel (CPOA) by crosslinking formylphenylboronic acid (FPBA)-grafted carboxymethyl chitosan with oxidized alginate and loaded with mesenchymal stem cell exosomes loaded with mesenchymal stem cell exosomes (CPOA@Exos) for diabetic wound healing. The hydrogel was injectable, self-healing, and responsive to reactive oxygen species, allowing both free radical scavenging and on-demand exosome release. In a diabetic mouse model, CPOA@Exos promoted M2 macrophage polarization, enhanced cell proliferation, and accelerated wound healing, offering a promising anti-inflammatory dressing for diabetic wounds.

Nucleic acid delivery, including plasmid deoxyribonucleic acid (pDNA), messenger ribonucleic acid (mRNA), and small interfering RNA (siRNA) represents another rapidly advancing frontier in polysaccharide-based biofunctionalization. Polysaccharides such as chitosan and its derivatives are particularly well suited for nucleic acid delivery due to their cationic nature, which enables electrostatic complexation with negatively charged nucleic acids. These complexes protect genetic cargo from enzymatic degradation and facilitate cellular uptake while reducing cytotoxicity compared to synthetic polycationic carriers. Incorporation of nucleic acid–loaded polysaccharide complexes into scaffolds allows spatial and temporal control over gene expression, enabling localized modulation of cell behavior during tissue regeneration [97]. For example, the chitosan-imidazole acetic acid formed nanocomplexes with nucleic acids, showed increased zeta potential and size, and achieved up to 100-fold higher pDNA transfection than native chitosan, comparable to polyethyleneimine (PEI). It also enabled efficient siRNA-mediated gene knockdown, demonstrating its potential as a safe and effective gene delivery carrier [200].

Nanotherapeutics, including polymeric nanoparticles, inorganic nanomaterials, and hybrid nanosystems, further expand the functional scope of polysaccharide scaffolds. These nanostructures can be embedded within polysaccharide matrices or chemically conjugated to scaffold components, enabling multistage delivery strategies and hierarchical control over therapeutic presentation. Polysaccharide-based encapsulation enhances nanoparticle stability, modulates release kinetics, and improves biocompatibility by shielding potentially reactive surfaces. When integrated into scaffold architectures, nanotherapeutics introduce localized nanoscale functionality without altering bulk mechanical properties [10, 162]. Biomimetic ECM nanostructures were created using layer-by-layer assembly of chitosan, oxidized alginate, and bovine serum albumin (BSA)-based nanoparticles. The nanoparticles formed porous structures, enabled sustained bone morphogenetic protein-2 (BMP-2) release, and maintained their activity. Glycine-arginine-glycine-aspartate-serine (GRGDS)-modified films promoted bone marrow stem cell adhesion and proliferation, and the combined presence of GRGDS and BMP-2 synergistically enhanced cell function and bone formation. These ECM coatings could improve the biocompatibility and bioactivity of various biomedical devices [201].

A critical design consideration for advanced payload delivery is the interplay between scaffold degradation, payload release, and biological response. As polysaccharide matrices undergo controlled degradation, encapsulated exosomes, nucleic acids, or nanoparticles are progressively released, enabling dynamic signaling environments that evolve with tissue remodeling [202]. Crosslinking density, charge distribution, and incorporation of secondary carriers can be tuned to synchronize payload release with regenerative timelines, minimizing burst release and off-target effects.

Despite their promise, delivery of advanced biological payloads presents challenges related to stability, dosage control, and regulatory complexity. Exosome heterogeneity, nucleic acid immunogenicity, and nanoparticle accumulation require careful material design and rigorous characterization. Polysaccharide-based scaffolds offer a modular and adaptable platform to address these challenges by integrating protective, degradable, and biointeractive features within a single material system. Collectively, the incorporation of exosomes, nucleic acids, and nanotherapeutics elevates polysaccharide scaffolds from passive delivery vehicles to active regulators of cellular communication and gene-level instruction. These advanced delivery strategies complement conventional bioactive molecule release and lay the groundwork for responsive, adaptive, and precision-guided regenerative systems discussed in the following sections.

### Responsive and adaptive polysaccharide systems

Responsive and adaptive polysaccharide systems represent an advanced class of biofunctional materials capable of dynamically adjusting their properties in response to local biochemical and physiological cues [203]. Unlike static scaffolds, these systems are designed to sense changes in the surrounding microenvironment and translate them into controlled structural, mechanical, or biochemical responses. Such adaptability enables scaffolds to more closely emulate native tissue behavior, where extracellular matrices continuously remodel in response to cellular activity, enzymatic signaling, and metabolic conditions. Polysaccharides are particularly well-suited for the development of responsive systems due to their inherent chemical versatility, degradability, and compatibility with reversible crosslinking mechanisms [204]. By incorporating stimulus-sensitive linkages or functional groups, polysaccharide scaffolds can be engineered to undergo controlled degradation, swelling, stiffening, or payload release in response to specific biological triggers. Among the most extensively explored stimuli are enzymatic activity, pH variation, and redox conditions, which are characteristic of dynamic regenerative and pathological environments.

#### Enzyme-, pH-, and redox-responsive materials

Enzyme-responsive polysaccharide materials exploit the presence of specific enzymes within the biological milieu to trigger scaffold degradation or functional transformation. Because enzymatic activity is closely associated with cell type, tissue state, and remodeling phase, enzyme-responsive systems enable highly localized and biologically relevant responses. Polysaccharides such as hyaluronic acid, chitosan, alginate, and dextran are naturally susceptible to enzymatic cleavage by hyaluronidases, lysozymes, and glycosidases, allowing degradation rates to be modulated by molecular weight, crosslinking density, and chemical modification [49]. Incorporation of enzyme-cleavable peptide linkers or labile bonds further enhances specificity and control over scaffold remodeling. For example, an intestine enzyme-responsive hydrogel was developed to improve oral delivery of hydrophobic imatinib. Methacrylic anhydride-modified carboxymethyl chitosan formed a 3D network that enabled enzyme-triggered, long-term drug release. Incorporating sodium deoxycholate enhanced intestinal permeability by opening tight junctions. The hydrogel achieved comparable in vitro cancer cell lethality to imatinib mesylate and, in vivo, showed sixfold greater tumor inhibition. This approach offers controlled release and improved oral bioavailability of hydrophobic anticancer drugs [63].

pH-responsive polysaccharide systems leverage variations in local acidity that arise during inflammation, ischemia, or cellular metabolism. Polysaccharides containing ionizable functional groups, such as carboxyl or amino moieties, undergo reversible changes in charge state in response to pH fluctuations, resulting in altered swelling behavior, solubility, or network integrity. Chitosan, in particular, exhibits pronounced pH sensitivity due to protonation of its amine groups under acidic conditions, enabling sol–gel transitions and controlled release behavior. Similarly, carboxylated polysaccharides demonstrate pH-dependent ionization that can be exploited to regulate hydrogel stability and permeability [205]. For instance, a novel polysaccharide-based composite hydrogel was developed as a biodegradable, pH-responsive adsorbent for selective removal of cationic (methyl green) and anionic (methyl orange) dyes. The hydrogel achieved high adsorption capacities (315 mg g⁻¹ for MG and 276 mg g⁻¹ for MO) and enabled separation of dye mixtures by pH. Adsorption followed the Freundlich isotherm and pseudo-second-order kinetics, was spontaneous and endothermic, and the hydrogel was reusable for at least ten cycles with good desorption efficiency. This simple, safe, and cost-effective material offers a sustainable approach for dye wastewater treatment [203].

Redox-responsive polysaccharide materials respond to variations in oxidative or reductive conditions, which are commonly associated with tissue injury, inflammation, and cellular signaling. Incorporation of redox-sensitive linkages, such as disulfide bonds, enables scaffold degradation or payload release in environments with elevated levels of reactive oxygen species or reducing agents. These systems provide a mechanism for coupling scaffold behavior to intracellular or inflammatory redox states, allowing selective activation in biologically active regions while maintaining stability under normal physiological conditions [139]. An important advantage of enzyme-, pH-, and redox-responsive systems is their ability to synchronize material behavior with biological processes. As cells infiltrate and remodel the scaffold, local enzymatic activity and metabolic byproducts dynamically regulate network degradation and bioactive molecule release. This feedback mechanism enables adaptive scaffolds to evolve alongside regenerating tissue, supporting early structural integrity while progressively yielding space and signaling cues for tissue formation. Redox-responsive dextran nanoparticles were developed by thiol modification to enable controlled release of hydrophobic molecules in response to cellular glutathione. The particles efficiently encapsulated hydrophobic dyes and drugs, with disulfide linkages cleaved under physiological conditions for rapid release. They were nontoxic to cells and, when loaded with paclitaxel, showed redox-triggered drug release and up to 90% inhibition of pancreatic cancer cells. These dextran-based carriers are versatile and promising for biomedical applications requiring controlled delivery of hydrophobic compounds [206].

Despite their promise, responsive polysaccharide systems require careful design to avoid premature activation or uncontrolled degradation. Sensitivity thresholds, response kinetics, and reversibility must be precisely tuned to ensure predictable performance across variable biological environments [207]. Integration of multiple responsive mechanisms within a single scaffold offers additional opportunities for hierarchical control but also increases design complexity. Overall, enzyme-, pH-, and redox-responsive polysaccharide materials represent a significant advancement in scaffold biofunctionality. By coupling material behavior to endogenous biological signals, these systems move beyond static support structures toward adaptive matrices capable of participating actively in tissue regeneration. Such responsiveness forms a critical foundation for more complex adaptive strategies, including microbiome-interactive systems and data-driven material design discussed in the following subsections.

#### Microbiome-interactive polysaccharide scaffolds

Microbiome-interactive polysaccharide scaffolds are currently best regarded as early-stage and context-specific design concepts rather than broadly established regenerative solutions. Nevertheless, they represent an emerging class of adaptive biomaterials designed to engage with resident or infiltrating microbial communities in a controlled and beneficial manner [208]. Rather than treating microorganisms solely as sources of infection, this paradigm recognizes the microbiome as an active participant in tissue homeostasis, immune regulation, and regeneration [209]. By leveraging the natural affinity between polysaccharides and microbial metabolic pathways, these scaffolds can be engineered to respond to microbial activity and, in turn, modulate host–microbe interactions within the regenerative microenvironment.

Polysaccharides are uniquely suited for microbiome-interactive design because many naturally occurring microbial species possess enzymatic machinery capable of degrading complex carbohydrates. Microbial glycosidases, polysaccharide lyases, and fermentation pathways enable selective metabolism of polysaccharide substrates, generating bioactive metabolites such as SCFAs that influence host immune responses and tissue repair processes [15]. By selecting specific polysaccharide compositions and architectures, scaffolds can be designed to preferentially support beneficial microbial populations while limiting pathogenic overgrowth.

Microbiome interaction can be achieved through both passive and active mechanisms. Passive interaction arises from the inherent biodegradability of polysaccharides, where microbial enzymatic activity contributes to scaffold remodeling and degradation. This process can introduce spatially and temporally regulated material breakdown that complements host-mediated enzymatic remodeling. Active interaction involves deliberate scaffold design to promote microbial colonization, metabolite production, or signaling. For example, incorporation of fermentable polysaccharide domains within a scaffold can encourage colonization by commensal microbes that produce anti-inflammatory metabolites, indirectly shaping the local immune environment [34].

The metabolites generated through microbial polysaccharide metabolism play a central role in microbiome-mediated biofunctionality. Short-chain fatty acids, including acetate, propionate, and butyrate, are known to regulate macrophage polarization, epithelial barrier function, and angiogenesis [210]. By enabling localized metabolite production within scaffold environments, polysaccharide materials can indirectly influence immune cell behavior and tissue remodeling without the need for direct delivery of immunomodulatory agents. This metabolite-driven mechanism complements the immune-instructive strategies described earlier and introduces an additional layer of biological regulation.

Scaffold architecture and fabrication strategy further influence microbiome interactions. Porosity, surface chemistry, and degradation rate affect microbial accessibility and colonization patterns. Highly porous or dynamically degrading polysaccharide networks may facilitate controlled microbial infiltration, whereas dense or rapidly crosslinked matrices may restrict microbial access [211]. These parameters must be carefully tuned to avoid unintended biofilm formation or excessive microbial proliferation, particularly in non-sterile or barrier-compromised tissues.

Despite their promise, microbiome-interactive scaffolds present unique challenges related to reproducibility, safety, and regulatory oversight. Microbial populations are inherently heterogeneous and influenced by host-specific factors, making standardized design and outcome prediction complex. Moreover, maintaining a balance between beneficial microbial engagement and infection risk requires precise control over scaffold composition, degradation kinetics, and local immune modulation [212]. As a result, microbiome-interactive strategies are currently best viewed as complementary components within broader regenerative designs rather than standalone solutions. A summary of representative polysaccharides, their microbial interactions, and the associated regenerative outcomes is provided in Table 4.

Overall, microbiome-interactive polysaccharide scaffolds expand the concept of responsive biomaterials by incorporating host–microbe interactions into scaffold design. By harnessing microbial metabolism and metabolite signaling, these systems introduce a novel and indirect pathway for regulating immune responses and tissue regeneration. This emerging strategy underscores the adaptability of polysaccharide materials and sets the stage for more predictive and data-driven design approaches discussed in the following section.

### Artificial intelligence and data-driven design of immune-instructive polysaccharide scaffolds

The increasing complexity of polysaccharide-based biomaterial systems has created a growing need for data-driven design approaches capable of integrating multiscale parameters across chemistry, fabrication, and biological performance. Traditional trial-and-error methods are often insufficient to navigate the vast design space defined by polysaccharide source, molecular modification, crosslinking strategy, scaffold architecture, and biofunctionalization. Data-driven and AI –assisted methodologies offer a systematic framework for identifying structure–function relationships, optimizing material properties, and accelerating the development of instructive polysaccharide biomaterials [198].

Machine learning and statistical modeling approaches have been increasingly applied to predict key material behaviors, including rheological properties, gelation kinetics, mechanical performance, and degradation profiles, based on input parameters such as molecular weight, functional group density, and processing conditions [17, 18]. In the context of polysaccharide scaffold systems, these tools enable rapid screening of formulation variables and fabrication parameters, reducing experimental burden while improving reproducibility. Predictive models are particularly valuable for extrusion-based bioprinting, where printability and shape fidelity depend on tightly coupled rheological and crosslinking properties.

Beyond materials processing, data-driven approaches are increasingly used to correlate scaffold architecture with biological outcomes. Multivariate analysis and supervised learning techniques can identify relationships between pore size, stiffness, degradation rate, and cellular responses such as proliferation, differentiation, or immune activation. By integrating experimental datasets with computational models, researchers can uncover non-intuitive design rules that guide scaffold optimization for specific biological functions without relying solely on empirical intuition [97, 198].

Artificial intelligence also supports the rational design of biofunctionalization strategies. Algorithms trained on datasets of ligand density, growth factor binding affinity, or immune response profiles can assist in selecting optimal combinations of polysaccharide chemistry and functional motifs [198]. In advanced delivery systems, data-driven models help predict release kinetics of bioactive molecules, exosomes, or nucleic acids based on scaffold composition and degradation behavior. These capabilities enable more precise control over spatiotemporal signaling within regenerative microenvironments. AI offers powerful tools for predicting biomaterial properties and reducing experimental cost; however, reliable prediction, particularly for complex biological outcomes such as bioactivity and cytotoxicity, remains strongly dependent on the availability of large, high-quality, and standardized datasets. Consequently, the widespread use of small, sparse, or heterogeneous data continues to be a major limitation and a primary cause of reduced model accuracy. Addressing these challenges will be essential for fully realizing the potential of AI, machine learning, and computational modeling in the design of next-generation polysaccharide-based materials [213].

**Table 4:** Microbiome-responsive polysaccharides and their regenerative outcomes

Polysaccharide	Microbial targets	SCFAs / Metabolites	Regenerative effect	Reference effect site		References
Inulin	Bifidobacteria	Butyrate	-Epithelial repair -Treg activation		Gut / systemic	[15, 214]
Pectin	Lactobacilli	Acetate, propionate	-Mucosal healing -Reduced IL-6		Gut / skin	[215]
Chitosan	Pathogen inhibition	Minimal	-Reduced biofilms -Enhanced re-epithelialization		Wound	[15, 216]
Alginate	Bacteroides spp.	Acetate	-Stem cell mobilization -Anti-inflammation		Gut / systemic	[15, 216]
Fucoidan	Unknown (marine)	Anti-pathogenic	-Angiogenesis, immune balance		Skin, oral mucosa	[217]

SCFAs, Short-chain fatty acids; IL-6, Interleukin-6

Importantly, the effectiveness of AI-assisted design depends on the quality and diversity of input data. Polysaccharide systems present unique challenges due to batch-to-batch variability, structural heterogeneity, and context-dependent biological responses. Standardization of material characterization, fabrication protocols, and biological assays is therefore essential to generate reliable datasets for model training [218]. Interdisciplinary integration of materials science, bioengineering, and computational expertise is a prerequisite for the successful implementation of data-driven design frameworks.

Despite these challenges, data-driven and AI-assisted approaches hold significant promise for advancing polysaccharide biomaterials toward predictive and personalized design. By enabling rapid iteration, reducing experimental uncertainty, and uncovering hidden structure–function relationships, these tools complement experimental strategies and support rational scaffold engineering. When integrated with responsive material systems and immune-instructive design principles, AI-assisted methodologies provide a useful platform for developing next-generation polysaccharide scaffolds tailored to specific regenerative contexts [219]. By enabling predictive control over chemistry, architecture, and signaling presentation, data-driven approaches provide a critical bridge between immune-instructive design principles and application-specific scaffold optimization. Table 5 compiles selected studies that exemplify the application of AI in the design and optimization of polysaccharide-based biomaterials.

**Table 5:** AI applications in polysaccharide-based scaffold research

AI technique	Polysaccharide type	Application	Outcome	References
Random forest	Alginate	Neural scaffold prediction	High accuracy in cell compatibility	[220]
Neural network	Chitosan	Bioprinting parameter optimization	Reduced structural error	[221]
Reinforcement learning	Hyaluronic acid	3D structure formation	Improved scaffold resolution	[169]
Support vector Machine	Dextran	Stem cell adhesion modeling	Predictive design of binding sites	[222]
decision trees	Fucoidan	Degradation rate prediction	Accurate bioresorption modeling	[223]

3D, Three-dimensional

## Applications in regenerative medicine

The versatility of polysaccharide-based scaffolds has enabled their application across a broad spectrum of regenerative medicine contexts. By integrating tunable mechanics, bioactivity, immune modulation, and advanced fabrication strategies, these materials have been adapted to address the distinct biological and structural requirements of diverse tissues. This section highlights representative applications in skin, musculoskeletal, cardiovascular, and neural regeneration, emphasizing how scaffold design principles translate into tissue-specific regenerative outcomes.

### Skin and wound healing

Polysaccharide-based tissue scaffolds have garnered significant attention for skin regeneration and wound healing due to their structural similarity to the ECM, excellent biocompatibility, and controllable biodegradability. These scaffolds provide a dynamic and supportive microenvironment conducive to cellular adhesion, proliferation, migration, and differentiation—key processes for re-epithelialization and dermal remodeling [224, 225]. Among polysaccharides, chitosan has been extensively explored in the form of films, electrospun fibers, and hydrogels for wound dressing applications. Its intrinsic antimicrobial properties, hemostatic potential, and positive surface charge facilitate cellular interactions and wound stabilization [226]. A notable study by Yan et al. [227] fabricated a 3D porous scaffold using a blend of chitosan, silk fibroin (SF), and hyaluronic acid via freeze-drying. In a full-thickness skin wound model in Sprague-Dawley rats, the SF/ hyaluronic acid / chitosan (85/5/15) scaffold exhibited superior dermal regeneration, enhanced angiogenesis, and improved collagen deposition compared to control groups. The scaffold also stimulated the secretion of critical growth factors such as platelet-derived growth factor (PDGF), VEGF, and basic fibroblast growth factors (bFGF), thereby accelerating tissue repair.

Hyaluronic acid -based scaffolds, due to their hydrophilicity and high water-retention capacity, contribute to a moist wound environment favorable for cell proliferation and migration. They are often used as temporary skin substitutes that not only protect the wound bed but also promote re-epithelialization and granulation tissue formation [228]. Furthermore, polysaccharide scaffolds can be chemically modified or functionalized to incorporate bioactive agents, such as antimicrobial compounds or growth factors, enhancing their therapeutic efficacy. Their tunable porosity supports oxygen and nutrient diffusion, while tailored degradation rates allow synchronization with tissue healing kinetics [229]. Current research continues to focus on optimizing crosslinking techniques, scaffold architecture, and surface functionalization to better mimic native dermal ECM and improve integration with host tissues [230]. Collectively, these advancements position polysaccharide-based scaffolds as versatile and effective platforms for advanced wound management and skin tissue engineering, offering significant potential for clinical translation in chronic wound care, burn treatment, and full-thickness skin replacement strategies.

### Bone and cartilage regeneration

Bone and cartilage regeneration present substantial structural and mechanical challenges because successful scaffolds must support tissue-specific cellular functions while providing appropriate mechanical stability, degradation kinetics, and biochemical cues. Polysaccharide-based materials have been widely investigated for musculoskeletal regeneration because of their generally favorable biocompatibility, tunable chemistry and degradation, high hydration capacity, and compatibility with reinforcing phases and bioactive components. However, many polysaccharide hydrogels lack the mechanical strength required for standalone load-bearing applications. Consequently, composite, hybrid, and interpenetrating-network designs are increasingly used to combine the biological advantages of polysaccharides with improved mechanical performance. Cartilage has limited intrinsic regenerative capacity because of its avascular nature and low cellularity, making effective repair of cartilage defects a continuing challenge in regenerative medicine [88]. Injectable polysaccharide-based hydrogels provide a minimally invasive platform capable of conforming to irregular defects and delivering cells or bioactive cues directly to damaged tissue. Chitosan, alginate, hyaluronic acid, and starch-based systems have been investigated because they can form highly hydrated matrices with tunable physicochemical properties and can be modified to support chondrocyte survival and ECM deposition [231]. Rather than uniformly reproducing the molecular composition of native cartilage ECM, these materials provide ECM-mimetic hydrated environments whose mechanical and biochemical properties can be tailored through polymer composition, functionalization, and crosslinking.

Hyaluronic-acid-based hydrogels have been investigated for their ability to support chondrocyte proliferation and ECM synthesis while providing a hydrated 3D microenvironment. Zhu et al. [67] reported favorable chondrocyte responses and integration using hyaluronic-acid-based hydrogel systems. Ansari et al. [232] developed a thermosensitive injectable hydrogel composed of glycerophosphate, chitosan, and starch that underwent in situ gelation at physiological temperature and supported chondrocyte viability and maintenance of the chondrogenic phenotype. Alginate-based hydrogels have likewise been extensively investigated for cartilage repair because of their high-water content, mild ionic gelation, and suitability for cell encapsulation. Hernández-González et al. [233] reported favorable cellular responses and tissue integration using injectable alginate-based systems. Because unmodified alginate does not possess strong intrinsic mammalian cell-adhesive motifs, biological performance can be further improved through functionalization or combination with cell-adhesive ligands, nanoscale materials, or synthetic polymers [234]. Composite hydrogels incorporating nanoparticles or bioactive ceramics have also been investigated to improve mechanical properties and support chondrogenic differentiation of MSCs [235].

**Fig. 3:**
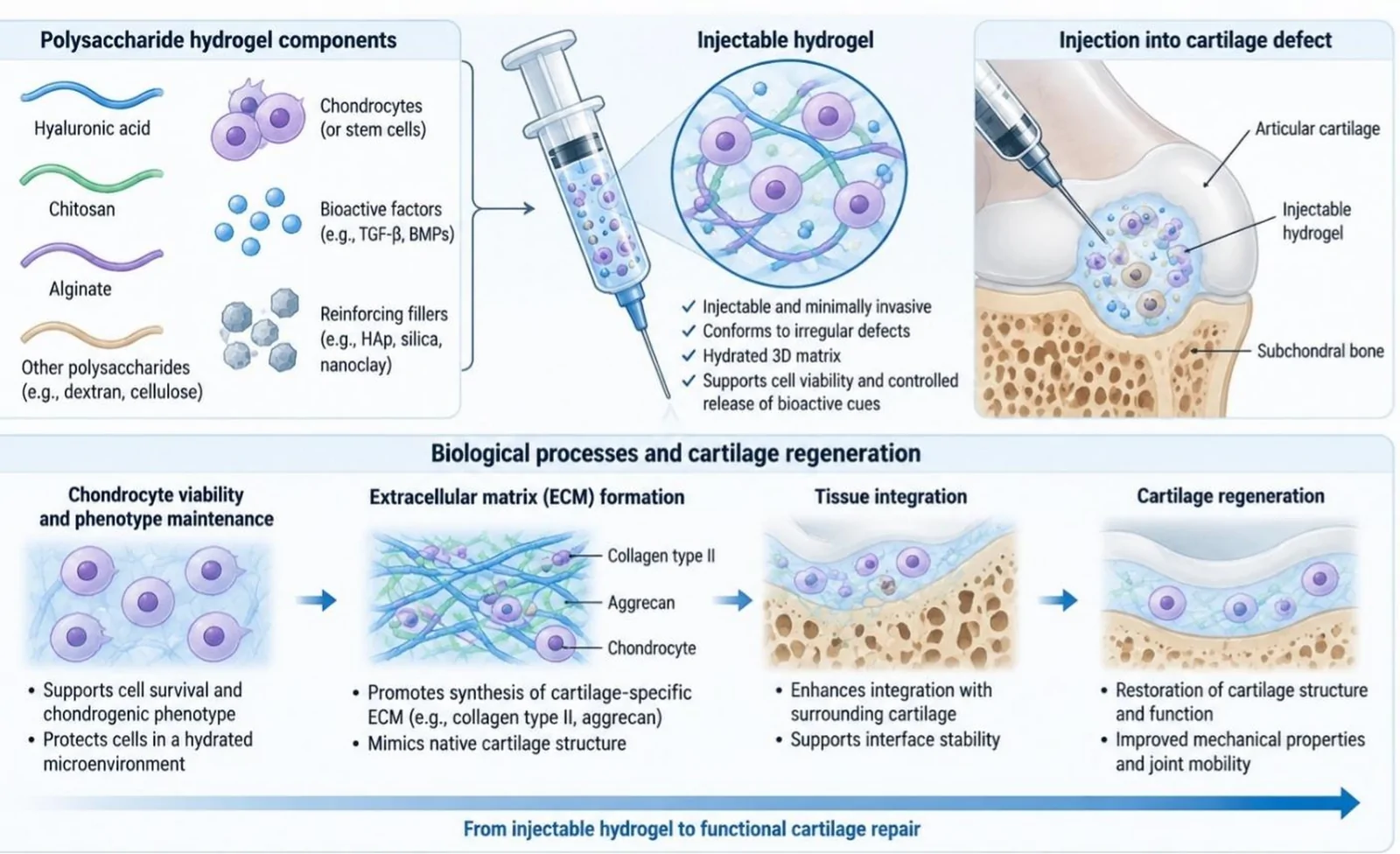
Injectable polysaccharide-based hydrogels for cartilage regeneration. Injectable hydrogels can conform to cartilage defects and provide hydrated three-dimensional matrices that support chondrocyte viability, extracellular matrix formation, and local tissue integration. TGF- β, transforming growth factor-beta; HAp, hydroxyapatite; BMP, bone morphogenetic protein; 3D, three-dimensional

Despite these advantages, important challenges remain, including control of in situ gelation kinetics, maintenance of mechanical stability under repetitive loading, avoidance of potentially cytotoxic or pro-inflammatory crosslinking residues, and coordination of scaffold degradation with cartilage matrix formation. Strategies including microgel incorporation, dual-network architectures, enzymatic crosslinking, and incorporation of nanoscale or ceramic components are therefore being explored to enhance hydrogel performance [236]. The principal functions, representative polysaccharides, and engineering strategies for injectable hydrogel-based cartilage repair are summarized in Fig. 3.

**Fig. 4:**
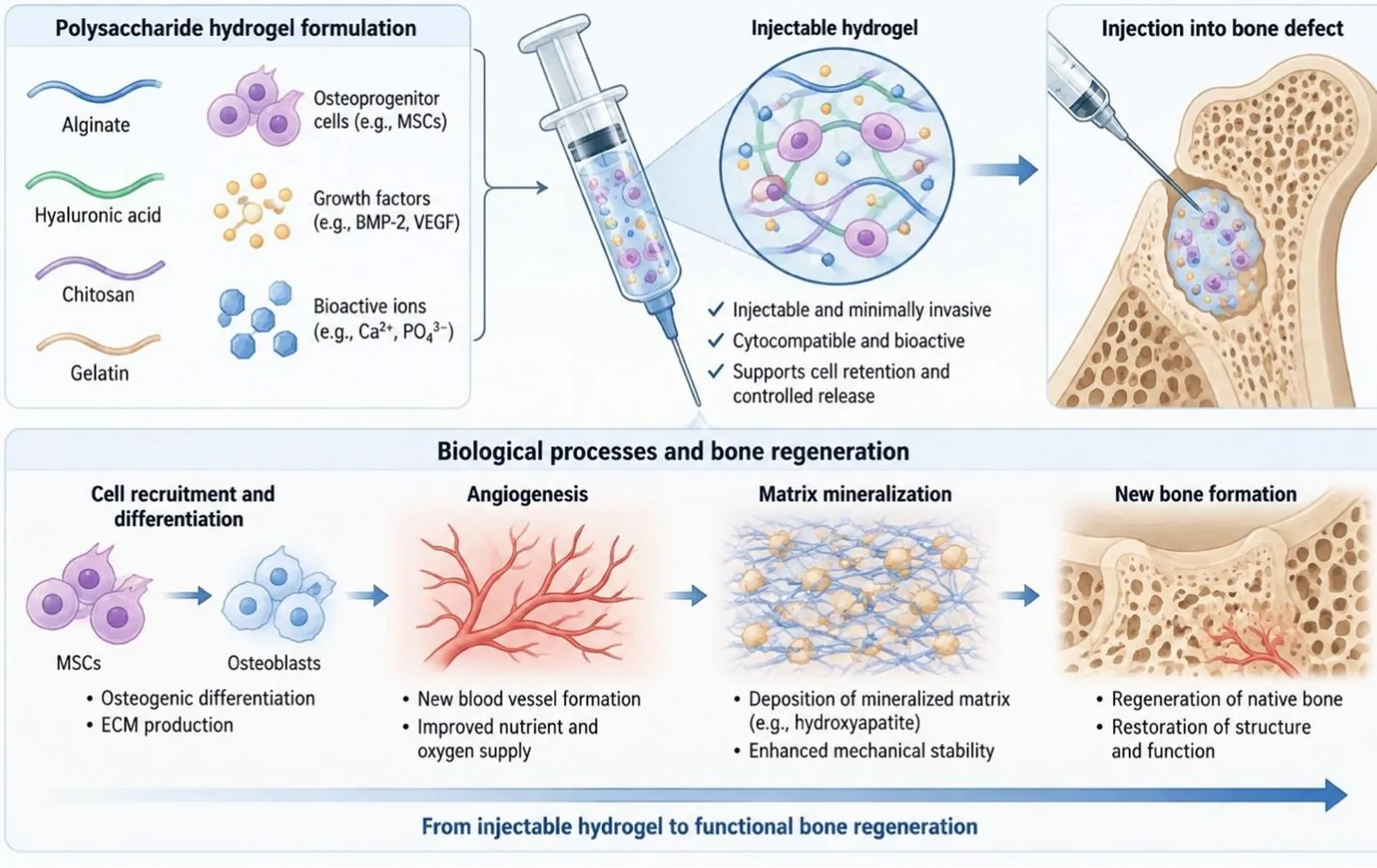
Injectable polysaccharide-based hydrogels for bone regeneration. Injectable polysaccharide hydrogels provide a three-dimensional matrix for local delivery of osteoprogenitor cells and bioactive cues while supporting matrix mineralization and new-bone formation. BMP-2, bone morphogenetic protein 2; MSCs, mesenchymal stem cells; VEGE, vascular endothelial growth factor; ECM, extracellular matrix

Injectable polysaccharide-based hydrogels are also being actively explored for bone regeneration because they enable minimally invasive administration, defect filling, in situ scaffold formation, and localized delivery of cells and osteogenic cues [237]. Hyaluronic acid, chitosan, alginate, dextran, and starch have been incorporated into bone-regenerative hydrogel systems either alone or, more commonly, as components of composite or hybrid matrices. Because polysaccharide hydrogels generally provide insufficient mechanical strength for major load-bearing bone defects, they are frequently reinforced with hydroxyapatite (HAp), bioactive glass, synthetic polymers, nanoparticles, or secondary polymer networks. They can also serve as delivery matrices for MSCs, osteoprogenitor cells, growth factors, and other osteoinductive agents. The principal design features and enhancement strategies for injectable polysaccharide-based hydrogels in bone regeneration are summarized in Fig. 4.

Hybrid polysaccharide-based systems illustrate how mechanical reinforcement can be integrated with biological functionality. Fang et al. [238] developed injectable hydrogels containing dextran, polyacrylamide, and HAp that exhibited increased compressive strength and osteoconductive properties and promoted bone regeneration in vivo. Similarly, El-Meliegy et al. [239] incorporated nano- HAp into dextran/chitosan composite scaffolds, improving Young’s modulus and compressive strength while supporting osteoblast proliferation and matrix mineralization. These studies demonstrate the importance of coupling polysaccharide matrices with reinforcing osteoconductive components when developing materials for mechanically demanding bone defects.

Hyaluronic-acid-based hydrogels have also been investigated for MSC attachment, proliferation, and osteogenic differentiation. Gilarska et al. [240] demonstrated that chemically modified hyaluronic-acid hydrogels containing osteoinductive cues supported bone regeneration in defect models. Chitosan-based systems are attractive because their polycationic character permits interactions with negatively charged biomolecules and mineral phases. Guillén-Carvajal et al. [226] reported that crosslinked chitosan hydrogels supported osteogenic differentiation of human stem cells while maintaining favorable mechanical and degradation characteristics. Alginate has similarly been employed in injectable bone scaffolds, particularly through calcium-mediated ionic gelation. Garske et al. [241] demonstrated delivery of MSCs and in situ matrix deposition using alginate hydrogels, while also highlighting the need for additional osteoinductive signals to obtain robust bone regeneration.

Accordingly, incorporation of osteogenic signals such as bone morphogenetic proteins (BMPs), bioactive glass, HAp nanoparticles, or other osteoconductive and osteoinductive components is being investigated to enhance the regenerative performance of polysaccharide-based systems. Functionalization with cell-adhesive motifs such as RGD peptides represents an additional strategy to improve cell attachment and lineage-specific differentiation [242]. Rather than functioning as passive carriers, these engineered hydrogels can therefore integrate mechanical support, cellular delivery, biochemical signaling, and controlled degradation within the defect microenvironment. Nevertheless, successful translation to critical-size bone defects will require further optimization of mechanical durability, degradation kinetics, vascularization, manufacturing reproducibility, and in vivo safety.

### Cardiovascular and vascular tissue engineering

Effective vascularization is critical for the survival, integration, and functionality of engineered tissues, particularly in large or metabolically demanding constructs [243]. Polysaccharide-based injectable scaffolds including chitosan, hyaluronic acid, and alginate are increasingly utilized to promote angiogenesis by mimicking the ECM, delivering pro-angiogenic cues, and supporting the formation of perfusable vascular networks [244]. These hydrophilic, biodegradable materials provide a conducive environment for cell migration, capillary infiltration, and tissue remodeling. Chitosan hydrogels loaded with VEGF, for instance, have been shown to enhance in vivo vascularization and tissue integration [245]. Hybrid scaffolds combining chitosan with synthetic polymers such as polycaprolactone (PCL) further improve endothelial recruitment and new vessel formation compared to PCL-only constructs [246]. Hyaluronic acid -based hydrogels, especially when functionalized with angiogenic peptides or ECM-mimetic motifs, support endothelial cell migration, tubular structure formation in vitro, and capillary infiltration in vivo [247]. Alginate hydrogels, crosslinked with calcium ions, can encapsulate endothelial progenitor cells and VEGF to enhance neovascularization and tissue perfusion, with tunable porosity enabling controlled release of angiogenic factors.

Emerging strategies include embedding nanoparticles or microspheres within polysaccharide hydrogels for sustained release of growth factors, as well as designing stimuli-responsive scaffolds that release angiogenic cues in response to pH or enzymatic activity [248, 249]. Such smart systems allow dynamic regulation of vascularization, improving integration while reducing unwanted neovascularization. These approaches are particularly valuable in regenerating complex tissues, including skin, bone, cardiac muscle, and composite organs, where timely vascularization is critical for successful regeneration.

Polysaccharide-based scaffolds have also been investigated for vascular tissue reconstruction, leveraging their high-water content, tunable mechanical properties, and ability to incorporate cells and bioactive factors [250]. Techniques such as hydrogel electrospinning create microfibrous templates that support ECM deposition and microvascular formation [251], while semi-interpenetrating gellan gum/ hyaluronic acid hydrogels promote neovascularization in ischemic models [252]. Encapsulation of endothelial cells within collagen/alginate-chitosan microcapsules facilitates 3D vascular network formation, lumen development, and cellular alignment, closely mimicking native vasculature [253, 254]. Polysaccharide-modified vascular grafts, such as PCL conduits functionalized with hyaluronic acid, enhance hydrophilicity, endothelial adhesion, and guided cell migration, supporting more effective vascular repair [255]. Overall, polysaccharide-based scaffolds provide versatile platforms for both promoting angiogenesis in engineered tissues and reconstructing vascular networks. Their modularity allows integration with cells, growth factors, and structural components, offering promising strategies for vascularized tissue constructs, small-diameter vascular grafts, and clinically translatable regenerative therapies. Future research is expected to focus on optimizing scaffold–host integration, perfusion in large constructs, and clinical translation of these vascular strategies.

### Neural and soft tissue regeneration

Polysaccharide-based scaffolds have attracted considerable interest for neural and soft tissue regeneration because of their generally favorable biocompatibility, biodegradability, high hydration capacity, and tunable physicochemical properties. Polysaccharides such as chitosan, alginate, hyaluronic acid, and dextran can be engineered into hydrated, ECM-mimetic scaffolds that provide structural support and permit incorporation of biochemical cues relevant to tissue repair [168, 256]. In neural tissue engineering, scaffold architecture, pore structure, surface properties, and fiber alignment can influence cell attachment, neurite extension, axonal guidance, and neural differentiation [257]. Polysaccharide-based hydrogels can also serve as delivery matrices for neurotrophic factors, stem cells, and other bioactive agents, thereby creating localized microenvironments that support neural-cell survival and regeneration [258].

**Fig. 5:**
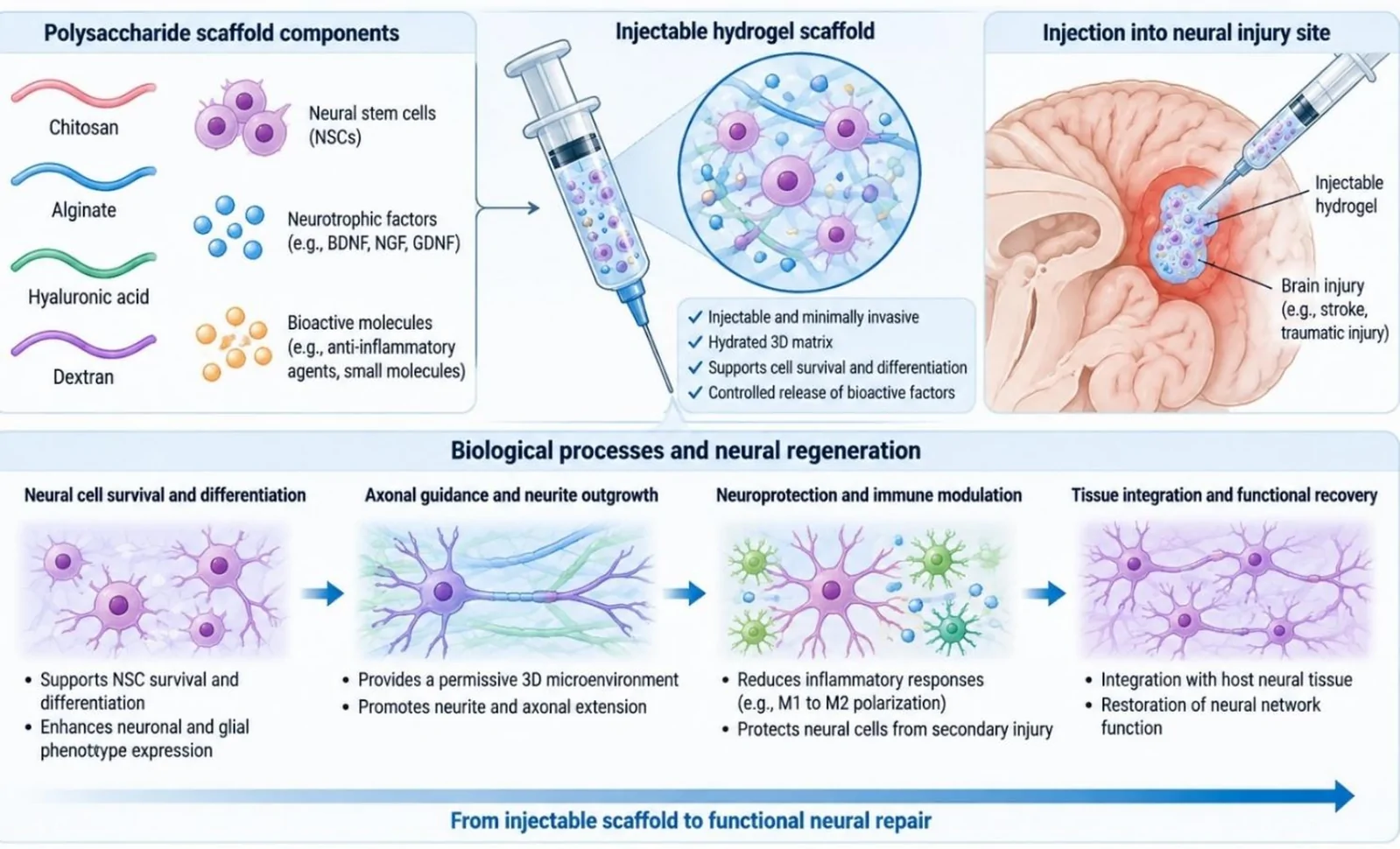
Injectable polysaccharide-based scaffolds for neural regeneration. Polysaccharide scaffolds based on materials such as chitosan, alginate, hyaluronic acid, and dextran can provide hydrated three-dimensional matrices for neural-cell delivery and local presentation of bioactive factors. NSC, neural stem cell. 3D, three-dimensional; NGF, nerve growth factor; GDNF, glial cell line–derived neurotrophic factor; BDNF, brain-derived neurotrophic factor

Injectable and self-healing polysaccharide hydrogels are particularly attractive for neural applications because they can be delivered with minimal surgical intervention and conform to irregular injury sites. Such systems may facilitate localized delivery of neural stem cells (NSCs) while providing a hydrated 3D matrix for cell survival and differentiation. Wei et al. [259], for example, reported that self-healing polysaccharide hydrogels maintained high NSC viability and enhanced expression of neuronal markers compared with conventional hydrogel formulations. Aligned fibers, neurotrophic-factor delivery, and shear-thinning or self-healing behavior can further be incorporated to support directional axonal growth and material retention after administration. These principal material functions and engineering strategies for polysaccharide-based neural scaffolds are summarized in Fig. 5.

**Fig. 6:**
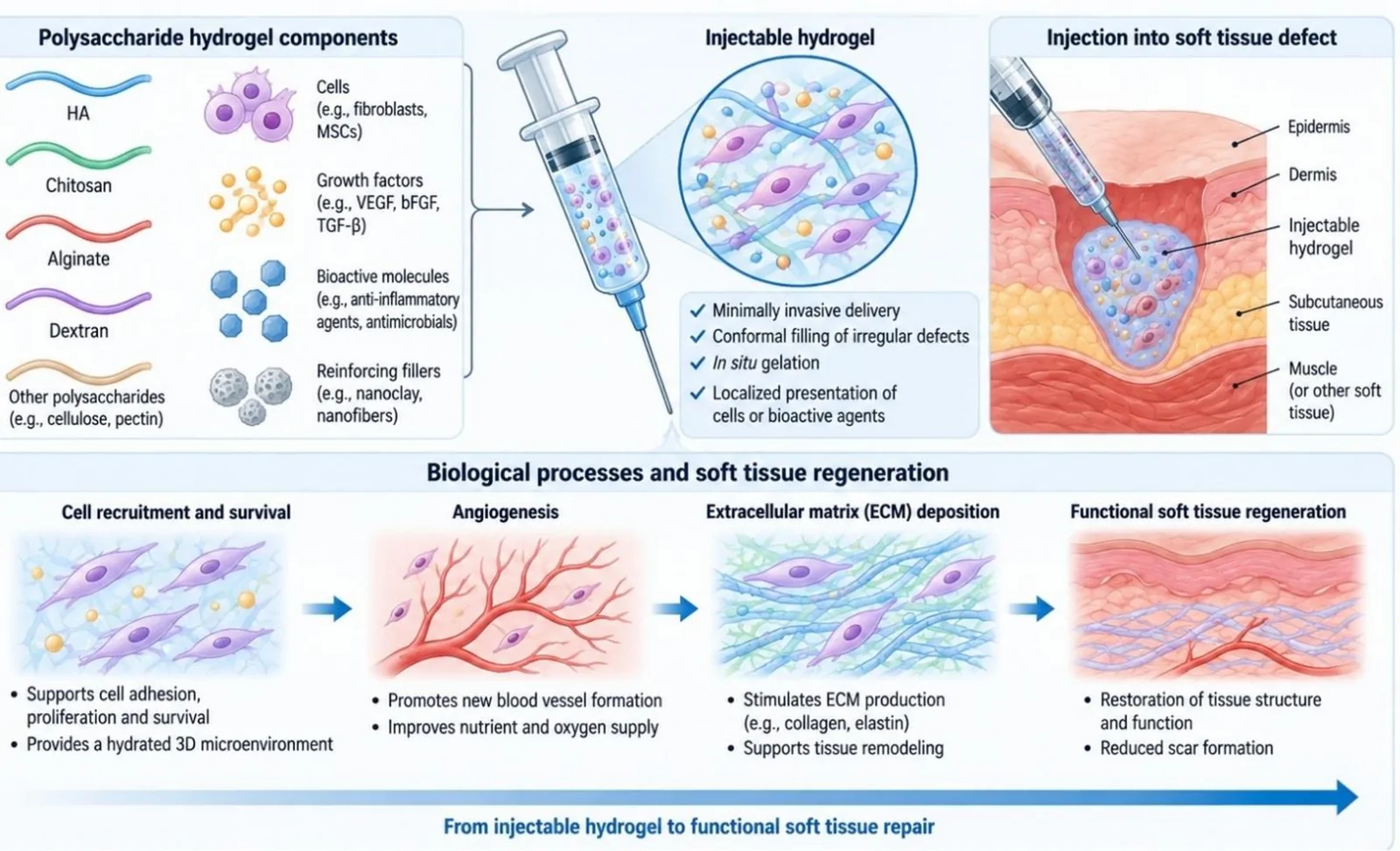
Injectable polysaccharide-based hydrogels for soft tissue regeneration. Injectable polysaccharide hydrogels can provide minimally invasive delivery, conformal filling of irregular defects, in situ gelation, and localized presentation of cells or bioactive agents. MSCs, mesenchymal stem cells; VEGE, vascular endothelial growth factor; TGF-β, transforming growth factor-beta; bFG, basic fibroblast growth factors; 3D, three-dimensional

Levan has also emerged as a microbial exopolysaccharide of interest for regenerative biomaterials because of its high water solubility, hydration capacity, reported cytocompatibility, antioxidant activity, and amenability to chemical functionalization. Catechol-conjugated levan has been investigated as a tissue-adhesive material and has demonstrated hemostatic and wound-healing activity in preclinical models [32]. In another approach, sulfated levan was incorporated into radially aligned PCL nanofibers for tympanic-membrane repair, illustrating how levan can be combined with structurally organized synthetic matrices to provide bioactive fibrous scaffolds [260]. These findings support the potential of levan as both a hydrated matrix component and a functional constituent of regenerative biomaterials, although its biological performance remains dependent on molecular modification, formulation, and application-specific design. Recent literature further highlights levan-based hydrogels as emerging platforms for wound healing and tissue-engineering applications [33]. This paragraph is important because it directly satisfies the reviewer’s request to include levan within the tissue-engineering applications section. Injectable polysaccharide-based materials also provide adaptable platforms for soft tissue regeneration because of their hydrophilicity, ability to conform to irregular defects, and capacity for localized delivery of cells and bioactive molecules [261]. Hyaluronic-acid-based injectable hydrogels have been investigated for dermal repair, where they can provide highly hydrated matrices that support fibroblast activity and neovascularization [259]. Dextran-based hydrogels prepared through click-chemistry approaches have likewise demonstrated cytocompatibility and regenerative potential in vivo [262]. Shear-thinning and self-healing polysaccharide hydrogels can further improve injectability and retention after administration, thereby supporting localized filling of irregular tissue defects [263]. Representative advantages, delivery mechanisms, and soft-tissue applications of injectable polysaccharide hydrogels are summarized in Fig. 6.

The regenerative performance of these scaffolds can be further enhanced through controlled delivery of cytokines, growth factors, and other pro-regenerative signals. Chitosan-based injectable systems, for example, have been reported to support axonal growth and Schwann-cell viability in peripheral nerve injury models [264]. In skin-repair applications, polysaccharide matrices can similarly serve as local depots for signals that promote fibroblast migration, angiogenesis, and matrix deposition. These effects arise from the combined influence of scaffold architecture, degradation, biochemical signaling, and host–material interactions rather than from polymer composition alone. Collectively, polysaccharide-based scaffolds provide versatile platforms for neural and soft tissue regeneration by integrating structural support, localized delivery, cell-instructive biochemical signals, and adaptable material properties. Future development should focus on improving control over degradation kinetics, mechanical matching with target tissues, reproducible manufacturing, long-term biological safety, and validation in clinically relevant injury models.

### Drug delivery systems

Polysaccharides are increasingly utilized in the fabrication of micro- and nano-scale drug delivery systems due to their excellent biocompatibility, structural versatility, and inherent capacity to interact with biological environments [265]. Their ability to form hydrogels, nanoparticles, micelles, or nanofibers enables encapsulation of a wide range of therapeutic agents, including small-molecule drugs, proteins, and nucleic acids. One key advantage of polysaccharide-based nanocarriers is their capacity to improve the solubility, stability, and bioavailability of poorly water-soluble drugs [30]. The polysaccharide matrix acts as a protective vehicle, minimizing premature degradation and enabling sustained or stimuli-responsive drug release [57]. Furthermore, due to their nanoscale size and surface characteristics, these carriers can exploit the enhanced permeability and retention effect to achieve passive targeting of tumors and inflamed tissues, improving therapeutic efficacy while reducing systemic toxicity.

Chemical functionalization of polysaccharides enhances their drug delivery capabilities. For instance, chitosan offers mucoadhesive and cell-penetrating properties, while hyaluronic acid can bind to CD44 receptors, enabling active targeting of cancer or inflamed cells [266]. Combining polysaccharides with other polymers via “grafting to” or “grafting from” reactions introduces tailored responsiveness to pH, redox potential, temperature, or enzymatic activity, providing precision in drug release timing and location [267]. Preclinical research has demonstrated significant promise. For example, a study by Gammon et al. [268] used an alginate-based nanocomposite scaffold to deliver cyclic dinucleotides and chimeric antigen receptor (CAR-T) cells in combination. This synergistic delivery system showed enhanced tumor regression, offering a compelling case for integrating immunotherapy and polysaccharide-based materials. However, translating such systems into clinical applications faces challenges, including drug burst release, nanocarrier aggregation, and regulatory hurdles [269]. Another notable example involves dextran-based hydrogels designed for sustained release of growth factors. Zhang et al. [270] developed VEGF–dextran– Poly (lactide- co-glycolide) [PLGA] microspheres, which significantly improved angiogenesis in a rat ischemic limb model, highlighting the role of polysaccharides in vascular tissue engineering.

Despite the growing number of successful in vitro and in vivo studies, most polysaccharide-based drug delivery systems remain in preclinical development. Their clinical translation will require further studies addressing scalability, reproducibility, and long-term biocompatibility under stringent regulatory frameworks. In summary, polysaccharide-based micro- and nano-fabricated drug delivery systems offer a highly adaptable and biocompatible platform for targeted and controlled therapeutic delivery. Continued progress in polysaccharide chemistry and fabrication methods will accelerate their application in oncology, tissue regeneration, immunotherapy, and pediatric drug delivery.

## Translational considerations and clinical outlook

Despite substantial advances in the design, biofunctionalization, and fabrication of polysaccharide-based scaffolds, their successful translation into clinically approved regenerative therapies remains contingent on overcoming a set of interconnected manufacturing, regulatory, biological, and economic challenges. While polysaccharides have demonstrated clear advantages in terms of biocompatibility, versatility, and biological relevance, translating increasingly complex scaffold systems from laboratory settings to clinical practice requires careful alignment of material innovation with translational feasibility. This section synthesizes key barriers to clinical implementation while outlining emerging strategies that may accelerate progress toward robust and scalable regenerative solutions.

### Scalability, manufacturing, and reproducibility

Scalable manufacturing and reproducible material performance are fundamental requirements for the clinical translation of polysaccharide-based scaffolds. Laboratory-scale fabrication commonly relies on tightly controlled processing conditions, customized formulations, and relatively small batch sizes, whereas translation to larger-scale manufacturing requires consistent raw materials, standardized processing, validated equipment, and reproducible quality-control procedures [271–273].Variability in polysaccharide source, extraction procedure, molecular-weight distribution, chemical modification, and purification can lead to batch-to-batch differences in mechanical behavior, degradation, and biological performance [274].

Raw-material sourcing represents a major contributor to this variability. Polysaccharides obtained from plants, algae, animals, fungi, or microbial biomass can exhibit compositional heterogeneity associated with species, geographic origin, harvest conditions, seasonal variation, and processing history [275]. Although this natural diversity contributes to structural and functional versatility, it can complicate standardization of molecular weight, substitution patterns, residual impurities, and biological activity. Microbial fermentation can provide greater control over production conditions and may improve batch consistency for selected polysaccharides; however, fermentation-derived products also require rigorous downstream purification and control of microbial contaminants [276, 277].

Manufacturing scalability is also strongly influenced by the selected fabrication strategy. Techniques such as freeze-drying, electrospinning, and solvent casting are widely used at laboratory scale, whereas large-scale production may require greater control of environmental conditions, process duration, solvent handling, equipment qualification, and product uniformity. Advanced fabrication approaches, including extrusion-based bioprinting and microfluidic processing, offer opportunities for automation and controlled processing, but reproducible scale-up remains dependent on standardized equipment, formulations, process parameters, and quality systems [272, 273]. Accordingly, variables such as polymer concentration, crosslinking conditions, temperature, shear exposure, pore-size distribution, and scaffold architecture should be controlled to maintain reproducible material and biological performance.

Integration of process analytical technology (PAT), in-line monitoring, and data-driven quality-control approaches may further improve manufacturing consistency. PAT frameworks are intended to strengthen understanding and control of manufacturing processes through timely measurement of critical material and process attributes, while quality-by-design approaches emphasize linking material attributes and process parameters to final product quality [278]. In polysaccharide-based scaffold manufacturing, real-time assessment of variables such as rheological behavior, crosslinking kinetics, dimensional fidelity, or related process outputs could therefore facilitate earlier identification of manufacturing deviations. Machine-learning-based models may further support analysis of multidimensional relationships among raw-material characteristics, processing conditions, and scaffold performance [279, 280].

Reproducibility must also encompass biological purity, particularly endotoxin burden. Natural and microbial polysaccharides may contain residual bacterial LPS, and variability in endotoxin contamination can alter inflammatory cytokine production and immune-cell responses even when the nominal polymer composition remains unchanged. Endotoxin burden should therefore be considered together with molecular weight, degree of substitution or deacetylation, residual protein and nucleic-acid content, ash or inorganic residues, and other physicochemical characteristics when defining the quality of biomedical-grade polysaccharides [281, 282]. Ravindranathan et al. [281] demonstrated that, within the chitosan samples examined, endotoxin content rather than degree of deacetylation or viscosity was the principal determinant of measured immunoreactivity.

Chitosan provides a particularly instructive example of why endotoxin control is essential. Reay et al. [282] showed that native chitosan preparations could contain sufficient endotoxin to induce pro-inflammatory responses and demonstrated that appropriate purification could markedly reduce endotoxin while preserving relevant chemical and degradation characteristics of the polymer. These observations indicate that apparently conflicting immunological effects reported for chitosan may partly reflect differences in purification and endotoxin burden rather than polymer chemistry alone. Consequently, endotoxin quantification and validated depyrogenation should be incorporated into material characterization when chitosan is intended for immunological evaluation or biomedical translation [281, 282].

Endotoxin control is similarly important for fermentation-derived microbial polysaccharides. Du et al. [277] investigated the high-molecular-weight exopolysaccharide GY785 produced by the Gram-negative bacterium *Alteromonas infernus* and demonstrated the presence of endotoxin-associated glycolipid contaminants in fermentation-derived preparations. They further showed that purification based on differential solubility in deoxycholate could substantially reduce these contaminants while preserving key molecular characteristics of the polysaccharide. This example illustrates why upstream fermentation, downstream purification, endotoxin reduction, and final-product testing should be treated as interconnected components of a biomedical manufacturing workflow.

Material variability must also be considered independently of endotoxin contamination. Lee et al. [62] demonstrated that chitosan immune responses are influenced by physicochemical and molecular-scale characteristics, helping to explain some of the divergent pro- and anti-inflammatory responses reported for different chitosan preparations. Lee et al. [24] further emphasized that the physicochemical and biological behavior of nanochitin and nanochitosan varies with molecular and nanoscale architecture. Thus, reproducibility requires simultaneous characterization of intrinsic material attributes—including molecular weight, degree of deacetylation, nanoscale organization, and chemical modification—and extrinsic quality variables such as endotoxin burden and residual processing contaminants.

For translational manufacturing, these parameters can be organized within a quality-by-design framework using critical quality attributes (CQAs) and critical process parameters (CPPs). ICH Q8 defines a CQA as a physical, chemical, biological, or microbiological characteristic that should remain within an appropriate range to ensure product quality, whereas a CPP is a process parameter whose variability can affect a CQA and therefore requires monitoring or control. For polysaccharide biomaterials, potential CQAs may include molecular-weight distribution, degree of substitution or deacetylation, residual reagents, protein and nucleic-acid contamination, endotoxin burden, crosslinking density, degradation kinetics, mechanical properties, and relevant biological activity. Corresponding CPPs may include extraction temperature and time, purification conditions, polymer concentration, crosslinking conditions, fabrication temperature, and shear exposure. Establishing experimentally validated relationships between these variables and final scaffold performance would strengthen manufacturing reproducibility and support regulatory translation [278]. Overall, successful clinical translation will require coordinated control of raw-material sourcing, extraction and purification, fabrication, biological purity, and final-product testing. Standardized manufacturing procedures, validated analytical methods, PAT-based process monitoring, clearly defined CQAs and CPPs, and robust endotoxin-control strategies can collectively reduce batch variability and improve the reproducibility and regulatory readiness of polysaccharide-based regenerative biomaterials.

### Sterilization, storage, and regulatory constraints

Sterilization, storage stability, and regulatory compliance are major considerations in the clinical translation of polysaccharide-based biomaterials. Many polysaccharides are sensitive to heat, irradiation, moisture, and chemical treatment; consequently, sterilization can alter molecular weight, crosslinking behavior, degradation, and mechanical properties. Steam sterilization may therefore be unsuitable for thermally sensitive systems, whereas gamma irradiation and electron-beam treatment can induce polymer-chain modification [283]. Chemical sterilization may provide a lower-temperature alternative, but residual sterilants must be carefully controlled. For biofunctionalized or cell-containing constructs that cannot tolerate terminal sterilization, validated aseptic processing may be required [278, 284]. Storage conditions likewise influence scaffold stability and biological performance. Lyophilization is frequently used to improve shelf life and facilitate storage of polysaccharide-based scaffolds; however, rehydration behavior, structural recovery, mechanical properties, and retention of biological activity should be validated after storage [285]. Stability assessment is particularly important for chemically modified scaffolds and systems containing growth factors, proteins, cells, or other sensitive bioactive components [286].

Regulatory requirements depend on the composition, intended function, and mechanism of action of the final product. Polysaccharide-based systems may be regulated as medical devices, combination products, or, when they contain substantially manipulated cells or tissue-engineered components, advanced therapy medicinal products, depending on the applicable jurisdiction and product characteristics [287, 288]. The diversity of regenerative and drug-delivery applications summarized in Table 6 further illustrates why sterilization, storage, manufacturing, and regulatory requirements must be defined according to the specific formulation and intended clinical use.

Endotoxin control represents an additional translational requirement, particularly for natural and microbial polysaccharides. Residual LPS can alter macrophage activation, cytokine production, and inflammatory responses, thereby confounding both safety assessment and interpretation of intended immunomodulatory effects [281, 282]. Accordingly, validated depyrogenation procedures and quantitative endotoxin testing should be incorporated into quality-control strategies, with acceptance criteria determined according to product type, administration route, dose, and applicable regulatory requirements rather than a single universal threshold [289]. Overall, successful translation of polysaccharide-based biomaterials requires coordinated consideration of sterilization compatibility, storage stability, biological purity, manufacturing consistency, and regulatory classification. Early definition of these requirements can reduce late-stage reformulation and improve the reproducibility, safety, and regulatory readiness of polysaccharide-based regenerative products.

### Clinical progress and persistent barriers

Polysaccharide-based biomaterials have achieved meaningful clinical presence in several regenerative contexts, particularly in wound care, dermal fillers, viscosupplementation, and hemostatic applications. Products based on alginate, hyaluronic acid, cellulose, and chitosan demonstrate that polysaccharides can meet safety and performance requirements when used in relatively simple formulations. Translation into more complex regenerative applications, including musculoskeletal, cardiovascular, and neural repair, has progressed more slowly. While many advanced scaffold systems show promising preclinical results, relatively few have advanced to late-stage clinical trials. This gap reflects the increasing complexity associated with integrating chemical modification, advanced fabrication, bioactive delivery, and immune modulation into a single platform. Each added layer of functionality introduces additional regulatory, manufacturing, and validation challenges. Demonstrating consistent and predictable clinical performance remains a major barrier. Regenerative outcomes are inherently multifactorial and patient-dependent, complicating trial design and endpoint selection. Variability in immune response, tissue condition, and defect characteristics can obscure scaffold-specific effects, often necessitating larger cohorts and longer follow-up periods. Economic considerations further influence adoption, as advanced scaffolds must demonstrate not only biological superiority but also practical advantages in cost, ease of use, and supply chain reliability.

**Table 6:** Representative applications of polysaccharide-based biomaterials in regenerative medicine and drug delivery

Regenerative application	Polysaccharides/materials	Scaffold/delivery format	Main biological function	Outcomes	References
Skin and wound healing	Chitosan, hyaluronic acid, SF, levan-based systems	Films, electrospun fibers, injectable or adhesive hydrogels, porous scaffolds	Cell support, angiogenic signaling, antimicrobial activity, extracellular-matrix remodeling, hemostasis, and tissue adhesion	Improved re-epithelialization, collagen deposition, vascularization, wound closure, and, in selected adhesive systems, wet-tissue adhesion and hemostatic performance	[32, 33, 225, 226, 228]
Cartilage regeneration	Chitosan, hyaluronic acid, alginate, starch	Injectable and thermosensitive hydrogels; composite and dual-network systems	Chondrocyte encapsulation and viability, extracellular-matrix synthesis, phenotype maintenance, and local mechanical support	Improved chondrocyte survival, cartilage-matrix formation, and integration with surrounding tissue in experimental models	[231–236]
Bone regeneration	Hyaluronic acid, chitosan, alginate, dextran, starch, HAp -containing composites	Injectable composite hydrogels and reinforced scaffolds	Osteogenic cell delivery, osteoconduction, matrix mineralization, and local mechanical reinforcement	Increased osteoblast activity, osteogenic differentiation, mineralized-matrix deposition, and new-bone formation in experimental models	[266, 238–242, 290]
Vascular tissue engineering	Chitosan, hyaluronic acid, alginate	Injectable hydrogels, microcapsules, vascular grafts	Endothelial-cell recruitment, delivery or retention of angiogenic factors, angiogenesis, and vascular-network formation	Increased endothelialization, capillary formation, vascularization, and tissue perfusion in experimental systems	[247, 251, 253, 291–294]
Neural regeneration	Chitosan, hyaluronic acid, alginate, dextran	Injectable, self-healing, and shear-thinning hydrogels; aligned fibrous scaffolds	Neural-cell survival, axonal guidance, neurotrophic-factor delivery, and neural differentiation	Improved neural-cell viability, neuronal-marker expression, neurite or axonal extension, and tissue integration in experimental models	[168, 258, 259, 264, 295]
Soft-tissue regeneration	Hyaluronic acid, dextran, chitosan, levan-containing systems	Injectable and self-healing hydrogels; adhesive or fibrous scaffolds	Defect filling, fibroblast support, angiogenesis, localized delivery, and tissue integration	Improved connective- and epithelial-tissue repair, neovascularization, matrix remodeling, and defect closure	[32, 259–261, 263, 264, 296]
Drug delivery	Chitosan, hyaluronic acid, alginate, dextran	Nanoparticles, hydrogels, microspheres, nanofibers	Drug protection, localized delivery, controlled release, and stimuli-responsive release	Improved therapeutic-agent stability, localized retention, controlled release, and, in selected systems, improved bioavailability or targeting	[30, 57, 265, 268, 297, 298]

### Future directions and translational opportunities

Despite these challenges, several converging trends offer promising pathways for advancing polysaccharide-based regenerative biomaterials. One major direction involves the development of adaptive and immune-informed scaffolds that dynamically respond to biological cues rather than relying on static designs. Integration of enzyme-sensitive, pH-responsive, redox-responsive, and microbiome-interactive elements may enable closer alignment with natural tissue remodeling processes. Advances in biofabrication are expected to further expand translational potential. Hybrid manufacturing strategies that integrate extrusion printing, microfabrication, and hierarchical assembly offer opportunities to address mechanical, transport, and biological requirements simultaneously. Data-driven and AI–assisted design frameworks are increasingly valuable for navigating the multidimensional design space of polysaccharide systems, enabling predictive optimization of chemistry, architecture, and signaling presentation. Equally important is the growing recognition that successful translation requires early and sustained integration of regulatory, manufacturing, and clinical considerations. Incremental translation strategies—where simpler polysaccharide systems are clinically validated before introducing additional complexity—are emerging as a pragmatic and effective pathway forward. Collaborative efforts between academic researchers, industry partners, and regulatory bodies will be essential for defining standardized benchmarks and accelerating adoption.

## Conclusions

Adaptive polysaccharide biomaterials have progressed from primarily structural matrices toward multifunctional systems in which molecular architecture, chemical modification, scaffold fabrication, and biological signaling can be deliberately integrated. This review establishes an engineering framework connecting polysaccharide source and structure with extraction and purification, functionalization, crosslinking, biofabrication, cell–material interactions, regenerative applications, and translational requirements. A central principle emerging from this framework is that biological performance cannot be attributed to polymer identity alone. Molecular weight, stereochemistry, glycosidic linkage, sulfation, chain conformation, chemical modification, scaffold mechanics, degradation, and interfacial protein adsorption collectively influence cellular and immune responses. Direct stereospecific glycan–receptor interactions should also be distinguished from indirect protein-mediated effects. Equally important, purification history and endotoxin burden must be considered when interpreting immunomodulatory activity, particularly for natural and microbial polysaccharides, because uncontrolled contamination can compromise mechanistic conclusions and experimental reproducibility. Continued progress will therefore require integration of material innovation with robust manufacturing and quality-control strategies. Key challenges include source and batch variability, standardized physicochemical characterization, scalable fabrication, validated depyrogenation, sterilization and storage stability, long-term biological evaluation, and product-specific regulatory requirements. Emerging approaches involving advanced biofabrication, computational modeling, AI systems biology, and data-driven process control may assist in addressing these challenges, but their translational value will depend on rigorous experimental validation and reproducible manufacturing. Future development should prioritize polysaccharide biomaterials whose complexity is justified by measurable biological benefit and whose molecular composition, mechanism of action, manufacturing process, and quality attributes can be reliably characterized and controlled. Integrating these requirements from early-stage design through preclinical evaluation may accelerate the development of safer, reproducible, and clinically translatable polysaccharide-based regenerative technologies.

## Data Availability

No datasets were generated or analysed during the current study.

## Abbreviations

3D: Three-dimensional
AI: Artificial intelligence
BDNF: Brain-derived neurotrophic factor
bFGF: Basic fibroblast growth factors
BMP-2: Bone morphogenetic protein-2
BSA: Bovine serum albumin
CAR-T: Chimeric antigen receptor
CCL22: Chemokine ligand 22
CD44: Cluster of differentiation 44
CPOA: Dual-dynamic covalent hydrogel
CPOA@Exos: Loaded with mesenchymal stem cell exosomes
CPPs: Critical process parameters
CQAs: Critical quality attributes
DNA: Deoxyribonucleic acid
DNase I: Deoxyribonuclease I
EAE: Enzyme-assisted extraction
ECM: Extracellular matrix
EDC: 1-ethyl-3-(3-dimethylaminopropyl) carbodiimide
Exos: Exosomes
FGFs: Fibroblast growth factors
FPBA: Formylphenylboronic acid
GDNF: Glial cell line–derived neurotrophic factor
GRGDS: Glycine-arginine-glycine-aspartate-serine
HAp: Hydroxyapatite
HB-EGF: Heparin-binding-epidermal growth factor
HRP: Horseradish peroxidase
HWE: Hot water extraction
IL-10: Interleukin-10
LMW: Low molecular weight
MAE: Microwave-assisted extraction
mRNA: messenger ribonucleic acid
MSCs: Mesenchymal stem cells
NET: Neutrophil extracellular traps
NGF: Nerve growth factor
NHS: N-hydroxysuccinimide
NSAIDs: Non-steroidal anti-inflammatory drugs
NSCs: Neural stem cells
PAT: Process analytical technology
PCL: Polycaprolactone
PDGF: Platelet-derived growth factor
pDNA: Plasmid deoxyribonucleic acid
PEI: Polyethyleneimine
PLGA: Poly (lactide- co-glycolide)
PMMA: Poly (methyl methacrylate)
PPARγ: Peroxisome proliferator-activated receptor gamma
RGD: Arginine–glycine–aspartic acid
RHAMM: Receptor for hyaluronan-mediated motility
RNA: Messenger ribonucleic acid
rtPA: recombinant tissue-type plasminogen activator
SCFAs: Short-chain fatty acids
SCPL: Solvent-casting particulate leaching
SF: Silk fibroin
siRNA: small interfering RNA
STAT6: Signal transducer and activator of transcription 6
TGF-β: Transforming growth factor-beta
TLR: Toll-like receptor
UAE: Ultrasonic-assisted extraction
USD: United states dollar
UV: Ultraviolet
VEGF: Vascular endothelial growth factor
